# Landscape of lipidomic metabolites in gut-liver axis of Sprague–Dawley rats after oral exposure to titanium dioxide nanoparticles

**DOI:** 10.1186/s12989-022-00484-9

**Published:** 2022-08-03

**Authors:** Zhangjian Chen, Shuo Han, Pai Zheng, Jiahe Zhang, Shupei Zhou, Guang Jia

**Affiliations:** 1 Department of Occupational and Environmental Health Sciences, School of Public Health, Peking University, Beijing, 100191 China; 2 Beijing Key Laboratory of Toxicological Research and Risk Assessment for Food Safety, School of Public Health, Peking University, Beijing, 100191 China; 3 Department of Laboratory Animal Science, Health Science Center, Peking University, Beijing, 100191 China

**Keywords:** Titanium dioxide nanoparticles, Nanotoxicity, Lipidomics, Lipid peroxidation, Gut-liver axis

## Abstract

**Background:**

The application of titanium dioxide nanoparticles (TiO_2_ NPs) as food additives poses a risk of oral exposure that may lead to adverse health effects. Even though the substantial evidence supported liver as the target organ of TiO_2_ NPs via oral exposure, the mechanism of liver toxicity remains largely unknown. Since the liver is a key organ for lipid metabolism, this study focused on the landscape of lipidomic metabolites in gut-liver axis of Sprague Dawley (SD) rats exposed to TiO_2_ NPs at 0, 2, 10, 50 mg/kg body weight per day for 90 days.

**Results:**

TiO_2_ NPs (50 mg/kg) caused slight hepatotoxicity and changed lipidomic signatures of main organs or systems in the gut-liver axis including liver, serum and gut. The cluster profile from the above biological samples all pointed to the same key metabolic pathway and metabolites, which was glycerophospholipid metabolism and Phosphatidylcholines (PCs), respectively. In addition, absolute quantitative lipidomics verified the changes of three PCs concentrations, including PC (16:0/20:1), PC (18:0/18:0) and PC (18:2/20:2) in the serum samples after treatment of TiO_2_ NPs (50 mg/kg). The contents of malondialdehyde (MDA) in serum and liver increased significantly, which were positively correlated with most differential lipophilic metabolites.

**Conclusions:**

The gut was presumed to be the original site of oxidative stress and disorder of lipid metabolism, which resulted in hepatotoxicity through the gut-liver axis. Lipid peroxidation may be the initial step of lipid metabolism disorder induced by TiO_2_ NPs. Most nanomaterials (NMs) have oxidation induction and antibacterial properties, so the toxic pathway revealed in the present study may be primary and universal.

**Supplementary Information:**

The online version contains supplementary material available at 10.1186/s12989-022-00484-9.

## Background

Nanotechnology referred to the science, engineering, and technology conducted at the nanoscale (1–100 nm) [1], which has been widely developed and applied in many fields such as medicine, environmental protection, food and agriculture, etc. [2]. Nanotechnology could benefit the food industry, but may also cause some adverse effects on consumers [3]. The increasing amount of nanomaterials added to the foods that people eat daily will be absorbed into the human body through the digestive tract and trigger health risks. Titanium dioxide nanoparticles (TiO_2_ NPs) are one of the most produced nanomaterials worldwide (on a mass basis) [4, 5]. Food-grade TiO_2_ (E171) comes with a wide particle size distribution, but the average particle size is smaller with the progress of technology [6, 7]. Recently, testing of food-grade TiO_2_ found that the mean particle size was 110 nm (30–400 nm) and up to approximately 36% of the particles were TiO_2_ NPs [6, 8]. A study showed that over 40% of TiO_2_ particles in commercial gums are TiO_2_ NPs, which can leach out and be swallowed when chewing [9]. Research has detected TiO_2_ NPs in human post-mortem liver and spleen, which emphasized that health risks due to oral exposure to TiO_2_ NPs cannot be excluded [10].

Due to small size, large surface area and many other special physicochemical properties, nanomaterials have a series of special biological characteristics, such as high absorption rate, low scavenging rate and high bioactivity [11–13]. Although a majority of biological processes occur at the nanoscale can enhance scientists’ work in medicine, imaging, chemical catalysis, food and agriculture [14, 15], the population and environmental health and safety (EHS) risks are also of concern [16–19]. Review of the toxicological data of TiO_2_ NPs via oral exposure demonstrated that the liver may be the target organ for adverse effects [20, 21]. However, the mechanism of liver toxicity has not been clarified. Acute oral exposure to TiO_2_ NPs at a very high dose (5 g/kg body weight) could accumulate in the liver and directly damage hepatocytes by triggering reactive oxygen species (ROS) and inflammation [22, 23]. However, considering that only approximately 0.02% to 0.1% of TiO_2_ NPs could be absorbed through the digestive tract and the rest were excreted through feces after long-term and low-dose oral exposure [24–27], TiO_2_ NPs may interact with gut microbiota, thereby affecting the gut-liver axis and indirectly causing hepatotoxicity [28]. Recently, the effect of TiO_2_ NPs on gut microbiota has been widely reported [29–32]. TiO_2_ NPs could destroy the integrity of intestinal bacteria and cause osmotic stress under dark conditions [33]. Alterations of gut microbiota can contribute to the pathogenesis of many disorders, including liver disease [34, 35].

Liver is the main metabolic organ of human body. Metabolic disorders are very sensitive to liver damage. Previous animal studies found that dietary intake of TiO_2_ NPs could induce glucose homeostasis imbalance [36, 37], including the disorder of glucose metabolism in liver. Meanwhile, pathological change of fatty degeneration in hepatocytes was found in rats after oral exposure to TiO_2_ NPs [38]. Other studies also indicated that lipid metabolism disorder may be one of the obvious toxic effects of TiO_2_ NPs [39–41]. The link between the gut microbiota and adipose tissue has been recently identified. It has been shown that bacterial product such as lipopolysaccharide (LPS) acts as a master switch to control adipose tissue metabolism [42]. Therefore, the changes of lipid metabolism in the gut-liver axis may be closely related to the hepatotoxicity induced by TiO_2_ NPs. In recent years, lipidomics provides a highly sensitive and high-resolution method for analyzing the structure and function of the complete set of lipids as well as their interactions [43]. It is sensitive enough to detect the subtle metabolomic changes when functional cellular assays showed no significant difference [40]. Research on lipid metabolism using lipidomics will contribute to the safety assessment of nanomaterials.

In the present study, we used lipidomics method to analyze the broad-spectrum lipophilic metabolites in liver, serum and feces of rats exposed to TiO_2_ NPs and compare to the control group by bioinformatics methods in detail. Changes of lipidomic signatures and key metabolites in gut-liver axis of rats induced by TiO_2_ NPs were analyzed. The possible mechanism of lipid metabolism change was also studied from the perspective of lipid peroxidation. We provided clues for better understanding oral toxicity of TiO_2_ NPs and the underlying molecular regulatory processes in vivo.

## Results

### Physicochemical properties of TiO_2_ nanoparticles

The physicochemical properties of TiO_2_ NPs were described in our previous work [28]. As shown in Table 1, the majority of the TiO_2_ NPs used in this study were spherical and anatase crystals with a purity of 99.90%. The average size of the TiO_2_ NPs measured by SEM was 29 ± 9 nm and the EDS combined with SEM confirmed that the atomic ratio of Ti and O was 1:2. The measured Brunauer–Emmett–Teller (BET) specific surface area of the TiO_2_ NPs was 77.51 ± 0.29 m^2^/g. The hydrodynamic diameter and zeta potential of TiO_2_ NPs (1 mg mL^−1^) in ultrapure water (H_2_O), artificial gastric juice (AGJ) and artificial intestinal juice (AIJ) were tested. TiO_2_ NPs tend to agglomerate into 40.8 ± 0.4, 149.3 ± 33.9, 168.1 ± 29.6 nm in H_2_O, AGJ and AIJ, respectively. The zeta potentials of TiO_2_ NPs in H_2_O, AGJ and AIJ were 11.09 ± 1.23, 4.73 ± 0.52 and − 13.7 ± 0.92 mV, respectively. The hydrodynamic size of TiO_2_ NPs in H_2_O, AGJ and AIJ were bigger than their primary size, which was likely due to the aggregation or agglomeration.

**Table 1:** Physicochemical properties of titanium dioxide nanoparticles (TiO_2_ NPs) used in the present study

Property	Method	TiO_2_ NPs
Shape	SEM	Spherical
Average diameter	SEM	29 ± 9 nm
Atomic percentage of Ti	EDS	32.66 ± 0.24%
Atomic percentage of O	EDS	67.34 ± 0.96%
Crystal structure	XRD	Anatase
Purity	ICP-MS	99.90%
Specific surface area	BET	77.51 ± 0.29 m^2^/g
Hydrodynamic diameter in H_2_O	DLS	40.8 ± 0.38 nm
Hydrodynamic diameter in AGJ	DLS	149.3 ± 33.9 nm
Hydrodynamic diameter in AIG	DLS	168.1 ± 29.6 nm
Zeta potential in H_2_O	DLS	11.09 ± 1.23 mV
Zeta potential in AGJ	DLS	4.73 ± 0.52 mV
Zeta potential in AIJ	DLS	− 13.7 ± 0.92 mV

SEM, scanning electron microscopy; EDS, Energy dispersive X-ray spectroscopy; XRD, X-ray powder diffractometry; ICP-MS, inductively coupled plasma mass spectrometry; BET, Brunauer–Emmett–Teller; H_2_O, ultrapure water; AGJ, artificial gastric juice; AIJ, artificial intestinal juice; DLS, dynamic light scattering

### Oral exposure to TiO_2_ nanoparticles affected lipid metabolism of liver in rats

**Fig. 1:**
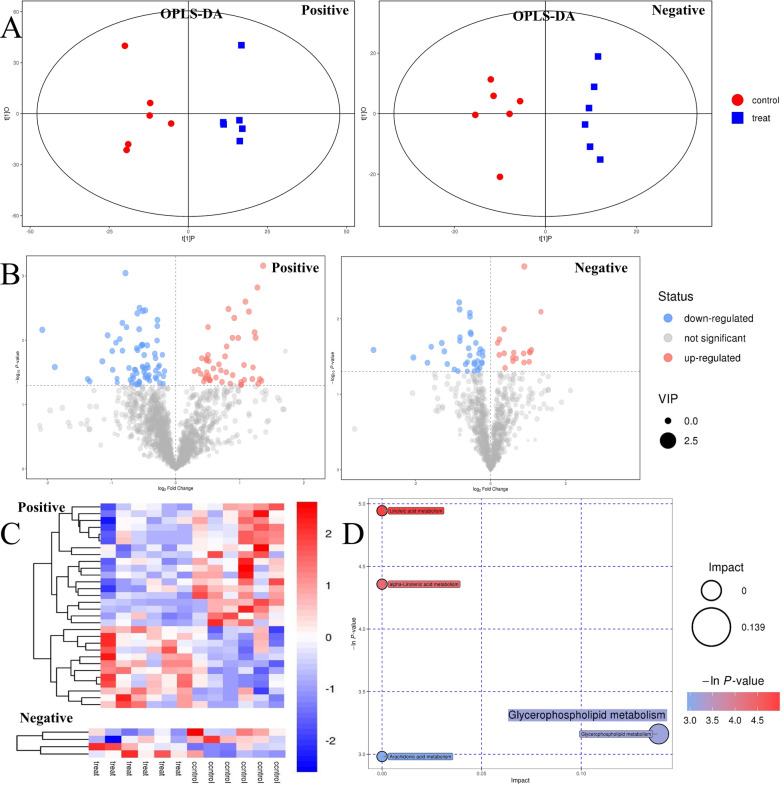
Effect of TiO_2_ nanoparticles on lipid metabolism in liver of rats. **A** Multivariate analysis of untargeted metabolomic data by OPLS-DA model showed the significant overall difference in metabolites of positive and negative ion modes between the TiO_2_ NPs treated group (50 mg/kg) and the control group. **B** Volcano plot was mapped to distinguish differential metabolites between different groups, taking VIP > 1 and *p* < 0.05 as the demarcation standard. **C** The concentrations of well-matched differential lipophilic metabolite in hepatic tissues were shown in heatmap and analyzed by hierarchical clustering, which revealed the change characteristics of metabolites among the experimental groups. **D** KEGG pathway analysis of differential metabolites between the control and treated group was shown in bubble chart. The names of significant pathways were labeled in the graph with *p* < 0.05 or Pathway Impact > 0.1. Glycerophospholipid metabolism pathway was significantly changed in liver by oral exposure to TiO_2_ NPs

Through the pathological observation and the detection of blood biochemical indexes related to liver injury, hepatotoxicity was observed in rats by oral exposure to TiO_2_ NPs at dose of 50 mg/kg BW for 90 days, which was reported in our previous study [28]. Fatty degeneration of hepatocytes was evident in the 50 mg/kg BW TiO_2_ NPs treated rats. In the present study, we further found lipidomic signatures of liver tissue in rats was changed after subchronic oral exposure to TiO_2_ NPs (50 mg/kg). As shown in Fig. 1A, multivariate analysis of metabolomic data by OPLS-DA model presented a clear separation trend between TiO_2_ NPs treated group (50 mg/kg) and the control group in both positive and negative ion modes, suggesting that there was a significant overall difference in metabolites between different groups. As shown in Fig. 1B, 2019 and 1030 lipophilic metabolites were identified from mass spectrometry data in positive and negative ion mode, respectively. Taking the variable importance in the projection (VIP) value of OPLS-DA model > 1 and p value from Student’s t-test < 0.05 as the criterion, 171 differential lipophilic metabolites (117 in positive ion mode and 54 in negative ion mode) between the liver samples in the control and TiO_2_ NPs (50 mg/kg) treated group were determined. Volcano plot was mapped to distinguish differential metabolites. Each point represents a metabolite. It was counted that 66 metabolites (48 in positive ion mode and 18 in negative ion mode) were significantly up-regulated and 105 metabolites (69 in positive ion mode and 36 in negative ion mode) were significantly down-regulated.

We further identified and matched the metabolites. Among 3049 lipophilic metabolites (2019 in positive ion mode and 1030 in negative ion mode) primarily identified, 644 metabolites (524 in positive ion mode and 120 in negative ion mode) were well matched from secondary mass spectrometry. Further analysis found that 34 well matched metabolites (30 in positive ion mode and 4 in negative ion mode) were differentially expressed in the control and TiO_2_ NPs (50 mg/kg) treated group, which were analyzed by hierarchical clustering and shown in heatmap (Fig. 1C). These differential metabolites could be clustered well. Among them, the concentrations of 14 metabolites including DG(18:2(9Z,12Z)/18:3(9Z,12Z,15Z)/0:0)[iso2], Pregnanetriol, TG(18:3(9Z,12Z,15Z)/20:3(8Z,11Z,14Z)/22:5(7Z,10Z,13Z,16Z,19Z))[iso6], and Ubiquinone 8, Lyso-, 1-(14-methyl-pentadecanoyl)-2-(8-[3]-ladderane-octanyl)-sn-glycerol and PC(22:6(4Z,7Z,10Z,13Z,16Z,19Z)/20:3(5Z,8Z,11Z)) et al. increased significantly compared with the control group. And 20 metabolites including PS(13:0/12:0), Nervonic Ceramide, TG(17:0/17:2(9Z,12Z)/17:2(9Z,12Z))[iso3], 20-Hydroxy-PGF2a, and [3-hexadecanoyloxy-2-[(4Z,7Z)-octadeca-4,7-dienoyl]oxypropyl] 2-(trimethylazaniumyl)ethyl phosphate et al. decreased significantly (Detailed information of differential metabolites was shown in Additional file 1: Table S1). KEGG pathway analysis of differential metabolites between the control and treated group was shown in bubble chart (Fig. 1D). Taking the negative natural logarithm of the p value (− ln (*p*)) > 2.996 as the criterion, 4 differential metabolic pathways were determined. After adjustment, only one metabolic pathway was statistically significant. Glycerophospholipid metabolism pathway (*p* < 0.05, Pathway Impact = 0.139) significantly changed in TiO_2_ NPs exposure group (Table 2). KEGG pathway map of glycerophospholipid metabolism was shown in Additional file 1: Figure S1.

**Table 2:** Metabolic pathways significantly affected in gut-liver axis of rats orally exposed to TiO_2_ NPs

Sample	Pathway name	*p*^*a*^	*FDR*^*b*^	Impact^c^	Hit metabolites^d^ (KEGG ID)
Liver	Glycerophospholipid metabolism	0.042	1.000	0.139	**PC (C00157)**
Serum	Glycerophospholipid metabolism	0.000	0.003	0.206	**PC (C00157)**; LysoPC(18:1(9Z)) (C04230); GlyceroPC (C00670)
Feces	Glycerophospholipid metabolism	0.000	0.036	0.164	**PC (C00157)**; PS(16:0/16:0) (C02737)

PC, Phosphatidylcholine; PS, Phosphatidylserine

^a^Primary *p* value of pathway enrichment analysis

^b^The *p* value was corrected by FDR

^c^The pathway impact was obtained by pathway topology analysis

^d^Name and KEGG ID of differential metabolites that hit the path

### Oral exposure to TiO_2_ nanoparticles affected lipid metabolism in serum of rats

In our previous study, we have found oral exposure to TiO_2_ nanoparticles affected lipid metabolism in serum of rats [39]. The level of triglycerides (TG) in rats administered to 10 mg/kg and 50 mg/kg TiO_2_ NPs daily for 90 days was significantly lower than the control group. Lipidomic signatures in serum of rats were also affected by subchronic oral exposure to TiO_2_ NPs (50 mg/kg). There were 456 differential lipophilic metabolites (343 in positive ion mode and 113 in negative ion mode) between the serum samples in the control and TiO_2_ NPs (50 mg/kg) treated group. Further analysis found that 70 well matched metabolites (69 in positive ion mode and 1 in negative ion mode) were differentially expressed in the control and TiO_2_ NPs (50 mg/kg) treated group (Detailed information of differential metabolites was shown in Additional file 1: Table S2). Glycerophospholipid metabolism pathway (*p* < 0.05, FDR = 0.003, Pathway Impact = 0.206) significantly changed in TiO_2_ NPs exposure group.

### Oral exposure to TiO_2_ nanoparticles affected lipid metabolism in gut of rats

**Fig. 2:**
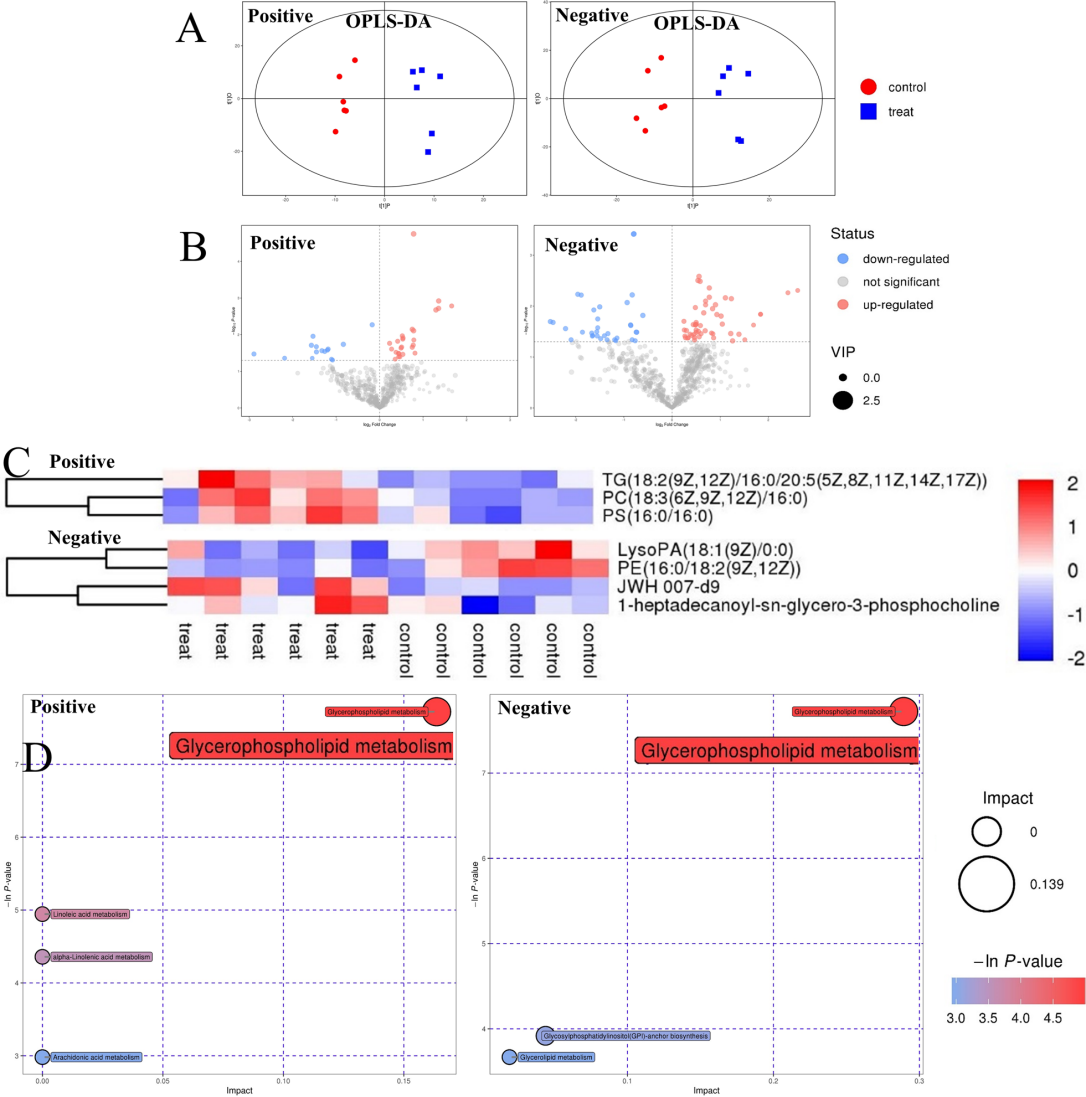
Effect of TiO_2_ nanoparticles on lipid metabolism in gut of rats. **A** Multivariate analysis of untargeted metabolomic data by OPLS-DA model showed the significant overall difference in metabolites of feces between the TiO_2_ NPs treated group (50 mg/kg) and the control group. **B** Volcano plot was mapped to distinguish differential metabolites between different groups, taking VIP > 1 and p < 0.05 as the demarcation standard. **C** The concentrations of well-matched differential lipophilic metabolite in feces were shown in heatmap and analyzed by hierarchical clustering. **D** KEGG pathway analysis showed that oral exposure to TiO_2_ NPs induced significant changes of Glycerophospholipid metabolism pathway in feces of rats

As the gut-liver axis may play an important role in hepatotoxicity induced by TiO_2_ NPs, lipid metabolism in gut also detected by fecal samples. We found TiO_2_ NPs indeed affected lipidomic signatures in gut of rats. As shown in Fig. 2A, the score map of OPLS-DA model suggested that there was a significant change in metabolites between different groups. As shown in Fig. 2B, 1811 and 1165 lipophilic metabolites were identified from mass spectrometry data in positive and negative ion mode, respectively. And 127 differential lipophilic metabolites (41 in positive ion mode and 86 in negative ion mode) between the fecal samples in the control and TiO_2_ NPs (50 mg/kg) treated group were determined. As shown in Fig. 2C, 7 well matched metabolites (3 in positive ion mode and 4 in negative ion mode) were differentially expressed in the control and TiO_2_ NPs (50 mg/kg) treated group (Detailed information of differential metabolites was shown in Additional file 1: Table S3). As shown in Fig. 2D, Glycerophospholipid metabolism pathway significantly changed in TiO_2_ NPs exposure group in both positive ion mode (*p* < 0.05, FDR = 0.036, Pathway Impact = 0.164) and negative ion mode (*p* < 0.05, FDR = 0.036, Pathway Impact = 0.289).

### Correlation and key metabolites of altered lipid metabolism in gut-liver axis of rats

**Fig. 3:**
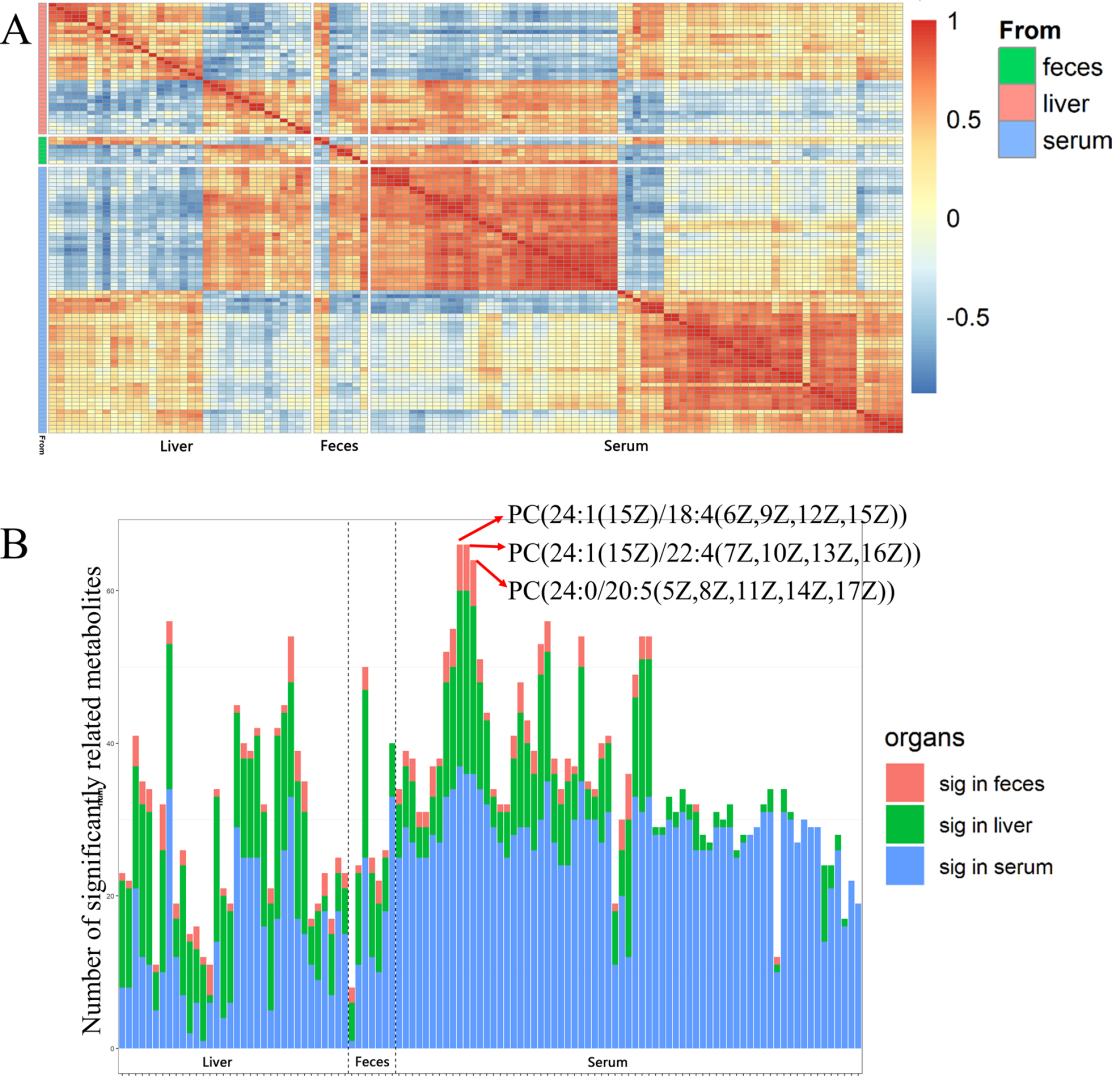
Correlation of lipophilic metabolites differentially expressed between the TiO_2_ NPs treated and control group in liver, serum and feces of rats. **A** Correlation analysis of differential metabolites from different sources showed that there was a wide correlation between them. **B** The number of significantly related metabolites (*p* < 0.05) between each metabolite and other metabolites was counted, according to three sources. It showed that the three metabolites with the most significant correlation with other metabolites were phosphatidylcholines (PCs)

In view of the lipid metabolism of main organs or systems in the gut-liver axis including liver, blood and gut was affected by TiO_2_ NPs, the correlation between them was further analyzed. Correlation analysis of altered lipophilic metabolites in liver, serum and feces of rats showed that there was a wide correlation between them (Fig. 3A). The number of significantly related metabolites of each metabolite from three sources was counted (Fig. 3B). All three metabolites with the most significant correlation with other metabolites were phosphatidylcholines (PCs), which were PC(24:1(15Z)/18:4(6Z,9Z,12Z,15Z)), PC(24:1(15Z)/22:4(7Z,10Z,13Z,16Z)) and PC(24:0/20:5(5Z,8Z,11Z,14Z,17Z)) in serum. It suggested that PCs may play an important role in the altered lipid metabolism of gut-liver axis induced by TiO_2_ NPs. Meanwhile, pathway analysis of differential metabolites showed that metabolic pathway significantly affected in liver, serum and feces were the same, which was glycerophospholipid metabolism pathway (Table 2). It indicted that the altered lipid metabolism of main organs or systems in the gut-liver axis was closely related, as the major changes were similar. Among hit metabolites, PCs (KEGG ID: C00157) were included in all three kind of samples (Table 2). It confirmed that PCs should be the key metabolites of altered lipid metabolism in gut-liver axis.

### Quantitative analysis of phosphatidylcholines (PCs)

**Fig. 4:**
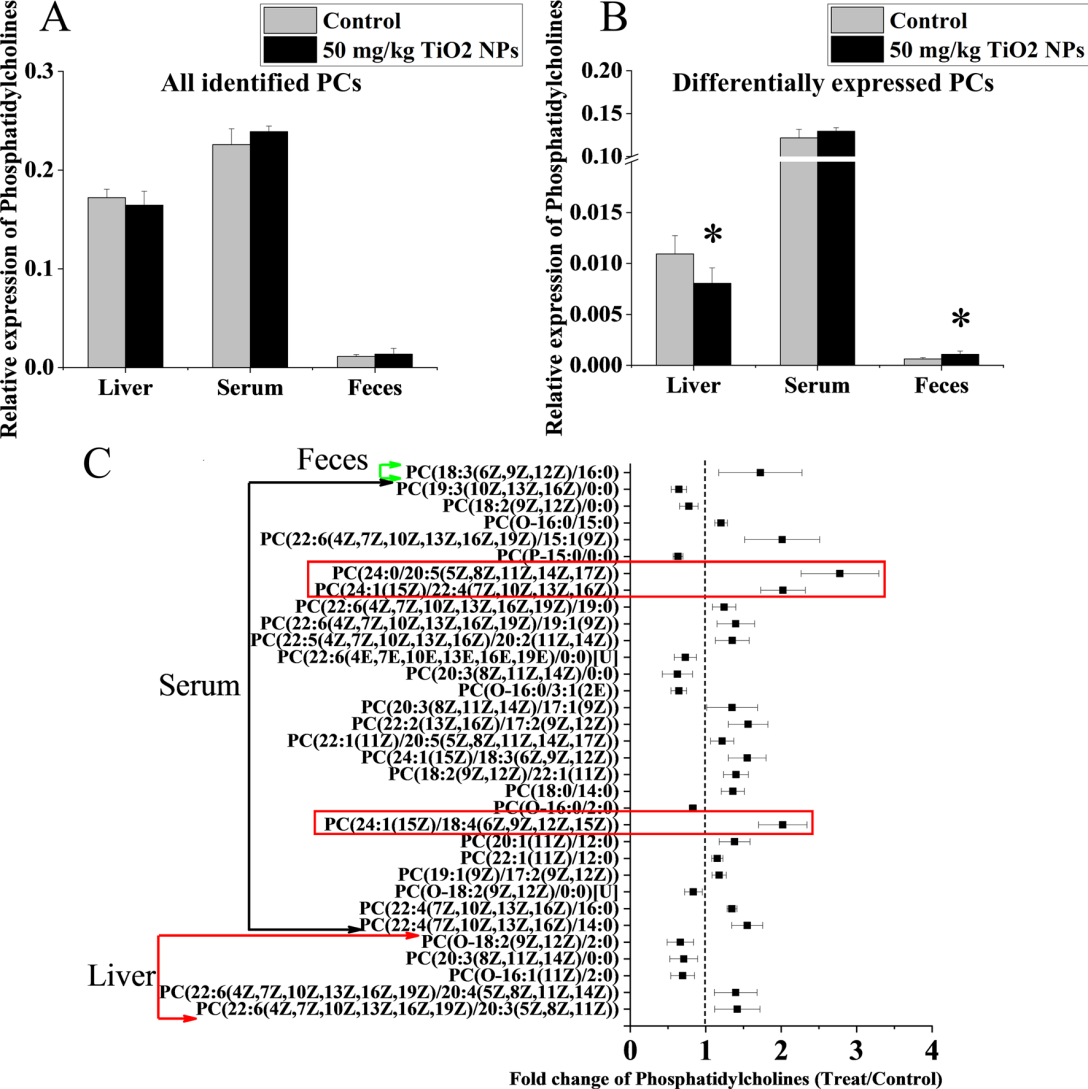
Relative expressions of phosphatidylcholines (PCs) in liver, serum and feces of rats after oral exposure to TiO_2_ NPs. **A** Total relative expression of all identified PCs from different sources showed no significant difference between the 50 mg/kg TiO_2_ NPs treated group and the control group. **B** Total relative expression of differentially expressed PCs significantly increased in feces and decreased in liver. **C** Fold changes of differentially expressed PCs between the 50 mg/kg TiO_2_ NPs treated group and the control group. The three PCs with the most significant correlation with other metabolites were labelled in red box. The changes of PCs were mainly manifested in some subclasses. Significant difference compared with the control group (∗ *p* < 0.05)

**Fig. 5:**
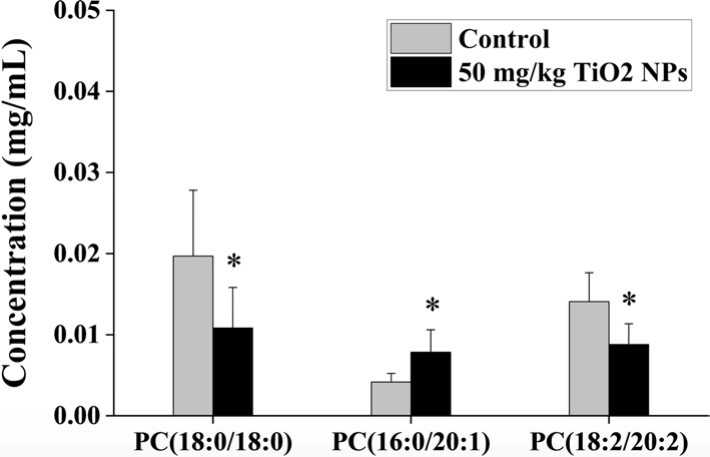
Absolute quantitative lipidomics verified changes of the concentrations of PCs in serum. Compared with the control group, the concentration of three kinds of PCs in serum of the 50 mg/kg TiO_2_ NPs treated group changed significantly. PC(16:0/20:1) increased significantly, while PC(18:0/18:0) and PC(18:2/20:2) decreased significantly. Significant difference compared with the control group (∗ *p* < 0.05)

Further collation of mass spectrometry (MS) data found that the total amount of PCs (all identified) did not change significantly, but the content of some PC subtypes with different MS parameters changed (Fig. 4). In feces, liver and serum, there was one kind, five kinds and twenty-seven kinds of PCs subtypes expressed differently between the TiO_2_ NPs treated group and the control group, respectively (Detailed information of differential metabolites was shown in Additional file 1: Table S4). The results suggested that we need to pay attention to the subtypes of PC, not just the total content of PCs, which may play a key role in the biological effects of oral exposure to TiO_2_ NPs. The total amount of differentially expressed PCs in liver decreased significantly, but in feces increased significantly (Fig. 4B). Among all differentially expressed PC subtypes, most of them increased in serum samples after treatment of TiO_2_ NPs, including the three kinds of PCs that had the most significant correlation with other metabolites (Fig. 4C). One subtype labelled as PC(20:3(8Z,11Z,14Z)/0:0) overlapped in serum and liver, and its content decreased both in serum and liver. In addition, we used absolute quantitative lipidomics to verify the changes of the concentrations of 48 kinds of PCs. It was found that the concentration of PC(16:0/20:1) increased significantly and the concentrations of PC(18:0/18:0) and PC(18:2/20:2) decreased significantly in the serum samples after treatment of TiO_2_ NPs (50 mg/kg) (Fig. 5).

### Lipid peroxidation in gut-liver axis

**Fig. 6:**
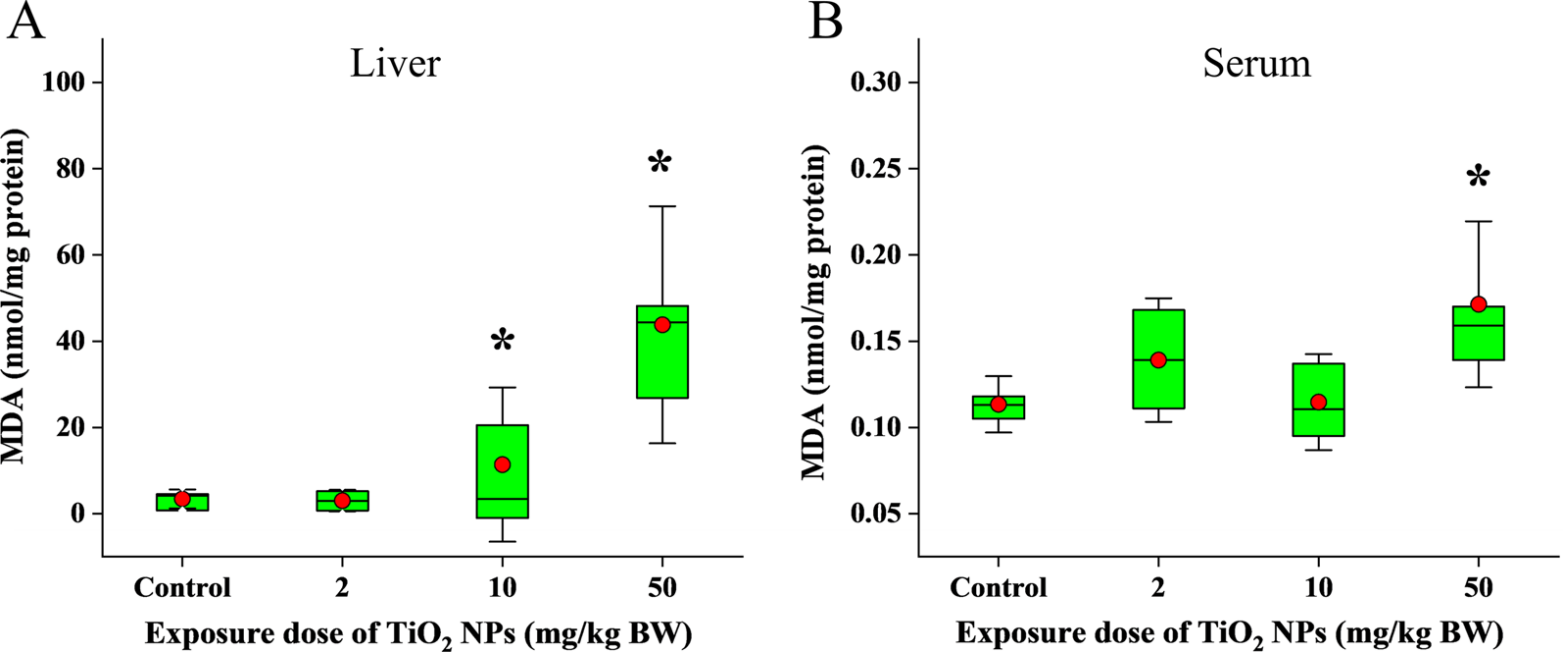
Lipid peroxidation induced by oral exposure to TiO_2_ NPs. **A** The concentration of malondialdehyde (MDA) increased significantly in liver tissue of rats in the TiO_2_ NPs treated groups (10 and 50 mg/kg) compared to the control group. **B** The concentration of malondialdehyde (MDA) also increased significantly in serum of rats in the TiO_2_ NPs treated groups (50 mg/kg)

**Fig. 7:**
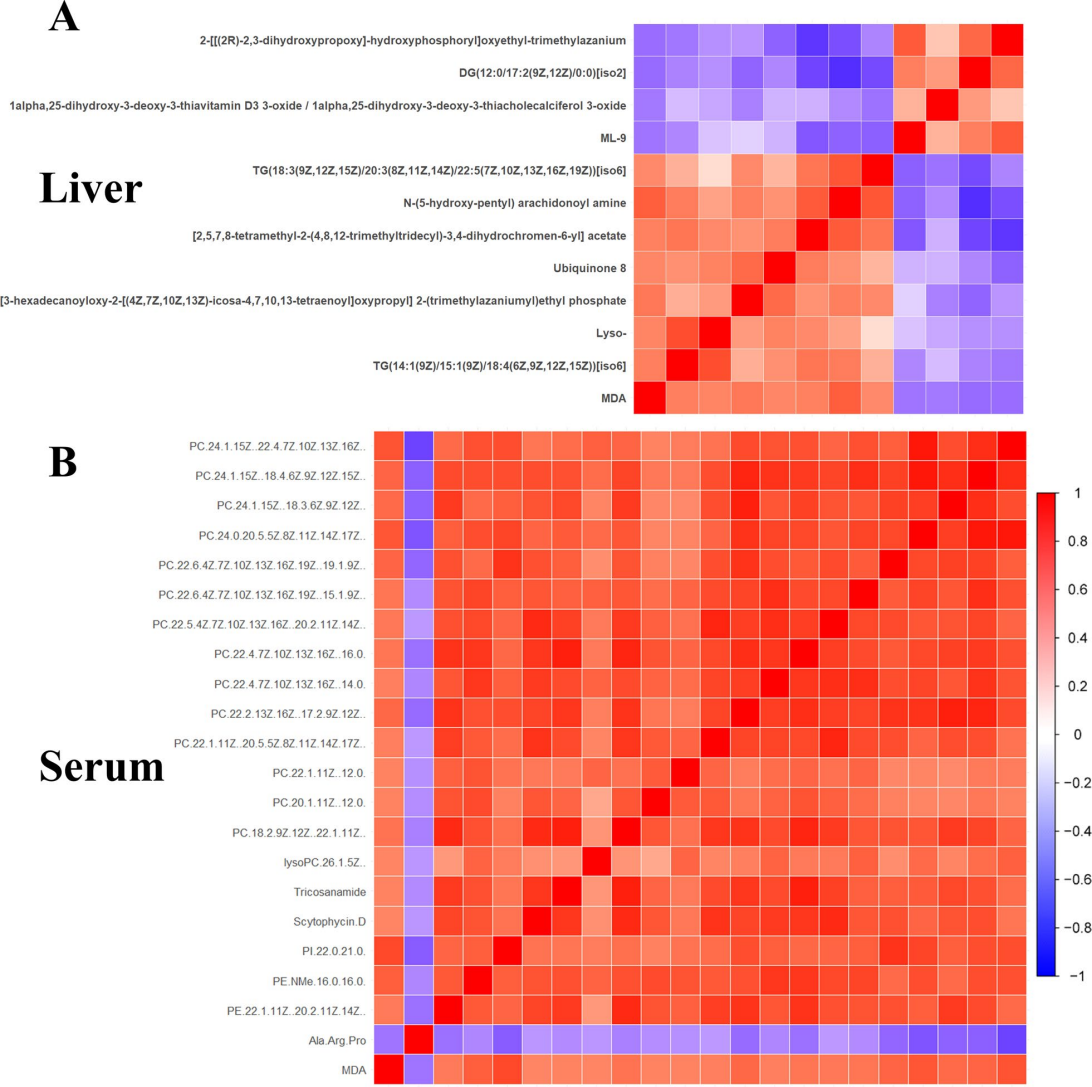
The correlation between the level of MDA and differential metabolites induced by oral exposure to TiO_2_ NPs in serum and liver. **A** MDA was significantly correlated with 11 kinds of differential metabolites in liver (*p* < 0.05), 7 of which were positively correlated. **B** MDA was significantly correlated with 21 kinds of differential metabolites in serum (*p* < 0.05), 20 of which were positively correlated. Lipid peroxidation was closely related to abnormal lipid metabolism induced by oral exposure to TiO_2_ NPs

Considering the strong oxidative induction ability of TiO_2_ NPs, lipid peroxidation may be one of the important mechanisms of lipid metabolism disorder. The increased concentration of malondialdehyde (MDA), representing a typical lipid peroxide, was observed in liver tissue of rats in the TiO_2_ NPs treated groups (10 and 50 mg/kg) compared to the control group (Fig. 6A). The concentration of malondialdehyde (MDA) also increased significantly in serum of rats in the TiO_2_ NPs treated groups (50 mg/kg) (Fig. 6B), indicating that the level of serum lipid peroxidation increased. Meanwhile, we examined the correlation between the level of MDA and differential metabolites in liver and serum (Fig. 7). Among 34 kinds of differential metabolites in liver, the relative expressions of 11 kinds were significantly correlated with the concentration of MDA in liver (*p* < 0.05), 7 of which were positively correlated. Among 70 kinds of differential metabolites in serum, the relative expressions of 21 kinds were significantly correlated with the concentration of MDA in liver (*p* < 0.05), 20 of which were positively correlated. Interestingly, among the 20 serum metabolites positively correlated with MDA, 14 metabolites were PCs. There may be a relationship between PCs and lipid peroxidation. So we thought that there was a close relationship between lipid peroxidation and abnormal lipid metabolism induced by oral exposure to TiO_2_ NPs.

## Discussion

In the present study, we focused on the effect of dietary intake of TiO_2_ NPs on lipid metabolism in gut-liver axis for the first time. Although there were still some inconsistent results from previous studies, liver was considered as the target organ for oral toxicity of TiO_2_ NPs [20]. Considering that the gut microbiota could be significantly affected by the ingestion of TiO_2_ NPs [29–32], the gut-liver axis, linking the intestine and liver, may be the key to inducing hepatotoxicity [28]. Furthermore, the effect of TiO_2_ NPs on lipid metabolism has been proposed and concerned recently. Both in vitro and in vivo metabolomics studies found that TiO_2_ NPs significantly affected the metabolic pathway of lipid metabolism [39, 40], which arose even in other metal oxide nanoparticles such as SiO_2_ and CeO_2_ nanoparticles. In this study, we used lipidomics to comprehensively understand the changes of lipid metabolism in the gut-liver axis induced by TiO_2_ NPs (Additional file 1: Figure S2). It was found that altered lipidomic signatures of main organs or systems in the gut-liver axis including liver, serum and gut were closely related, as the key metabolic pathway and lipophilic metabolites were the same. Glycerophospholipid metabolism pathway and PCs played an important role in the altered lipid metabolism of gut-liver axis induced by TiO_2_ NPs.

Glycerophospholipids are the main lipid constituents of cell membranes, which can directly affect the physiological functions of cells [44]. Changes in the glycerophospholipid metabolism pathway first prompted the transformations of cell membrane composition and permeability. Using a combination of fluorescent probes, Sohm et al. found that ingested TiO_2_ NPs could induce oxidative stress under dark conditions and cause intestinal bacteria membrane depolarization such as *Escherichia coli* and loss of integrity, leading to higher cell permeability [33]. In the present study, we found differentially expressed glycerophospholipids in feces, including phosphatidylcholine (PC) and phosphatidylserine (PS). Specifically, PC(18:3(6Z,9Z,12Z)/16:0) and PS(16:0/16:0) increased significantly in feces, which may be caused by bacterial fragmentation, increasing cell membrane components in feces. Alterations of gut microbiota can contribute to the pathogenesis of liver disease by affecting on host metabolism through intestinal-hepatic circulation and the gut-liver axis [34, 35, 45, 46]. In fact, the present study found four kinds of differentially expressed glycerophospholipids in gut-liver axis, including phosphatidylcholine (PC), LysoPC, GlyceroPC and phosphatidylserine (PS) (Table 2). Among them, PC altered significantly in all three kinds of samples including liver, serum and feces of TiO_2_ NPs-treated rats. So PCs may be the key metabolites in the effect of TiO_2_ NPs on the gut-liver axis and hepatotoxicity.

Phosphatidylcholine (PC, lecithin) is the most abundant glycerophospholipid in animal and plant tissues. The fluctuation of glycerophospholipid content will reflect the disorders of lipid metabolism and is an important biological indicator [47]. Liver is the major site of glycerophospholipid biogenesis and glycerophospholipids are important constituents of hepatocytes and mitochondrial membranes. It was reported that targeting of glycerophospholipid metabolism was a therapeutic approach that had been applied to several diseases [48]. Decreased hepatic phosphatidylcholine (PC) content had previously been linked to non-alcoholic fatty liver disease (NAFLD) [49]. The present study found that the total amount of differentially expressed PCs in liver decreased significantly. Meanwhile, pathological change of fatty degeneration in hepatocytes, which was similar to NAFLD, was also induced by oral exposure to TiO_2_ NPs. This further verified that PCs played an important role in the hepatotoxicity of TiO_2_ NPs.

The circulatory system, especially the portal vein, transports intestinal substances through the liver, which is an important place for intermediary metabolism of the gut-liver axis. In the present study, we confirmed the relationship between the changes of serum lipidomics and that in the gut and liver. The same key metabolic pathway (Glycerophospholipid metabolism) and metabolites (PCs) were found in analysis of serum lipidomics. Of course, the characteristics of serum metabolism are also the comprehensive performance of the whole body organ and system health effects. Therefore, we found metabolic changes in serum were more complex than that in liver and feces. For example, more differential metabolites including more subtypes of PCs were observed in serum and the most related three kinds of PCs (PC(24:1(15Z)/18:4(6Z,9Z,12Z,15Z)), PC(24:1(15Z)/22:4(7Z,10Z,13Z,16Z)) and PC(24:0/20:5(5Z,8Z,11Z,14Z,17Z))) were all in serum. In addition, pathway analysis also found that LysoPC and GlyceroPC differentially expressed in serum. Besides PC, LysoPC and GlyceroPC are important glycerophospholipids. Low level of LysoPC in plasma might be a general indicator of severity of malignant disease, such as cancer, and also correlated with weight loss and inflammatory parameters [50]. A prospective metabolomics study also found that higher plasma levels of lysoPCs were consistently related to lower risks of breast, prostate, and colorectal cancer [51]. Moreover, LysoPCs have been suggested to mediate anti-inflammatory effects in the liver as they negatively correlated with intrahepatic C-reactive protein [52, 53] and induced secretion of anti-inflammatory signals in human Tregs that lead to a reduction of macrophage migration [54]. Decreased GlyceroPC was also highly correlated with NAFLD [55]. Thus, we thought that the changes on serum lipidomic signatures induced by oral exposure to TiO_2_ NPs may be closely related to the hepatotoxicity. The changes of intermediate metabolism further confirmed the important role of gut-liver axis in the hepatotoxicity induced by TiO_2_ NPs.

The strong oxidative stress induction ability of TiO_2_ NPs was originally thought to be the main reason that TiO_2_ NPs can affect lipid metabolism after oral exposure. Elevated levels of oxidative stress are responsible for various adverse health effects, such as peroxidation of lipids and oxidative damage of DNA. It was widely reported that TiO_2_ NPs could produce excessive oxygen free radicals and ROS when interacting with biological tissue [56, 57]. In vitro, TiO_2_ NPs induced significant oxidative DNA damage and caused apoptosis in liver cells (HepG2) even at very low concentrations (1 µg/mL) [58]. In vivo*,* orally ingested TiO_2_ NPs also could generate excessive ROS by high accumulation of TiO_2_ NPs in the liver tissue and then triggered oxidative DNA damage and apoptosis through the intrinsic pathway, leading to hepatic injury in mice [59]. In the present study, we confirmed that oral exposure of TiO_2_ NPs increased levels of lipid peroxidation (MDA) in gut-liver axis including both serum and liver. Therefore, we believe that lipid peroxidation may be the initial step of lipid metabolism disorder, which should be partly responsible for oral toxicity induced by TiO_2_ NPs.

**Fig. 8:**
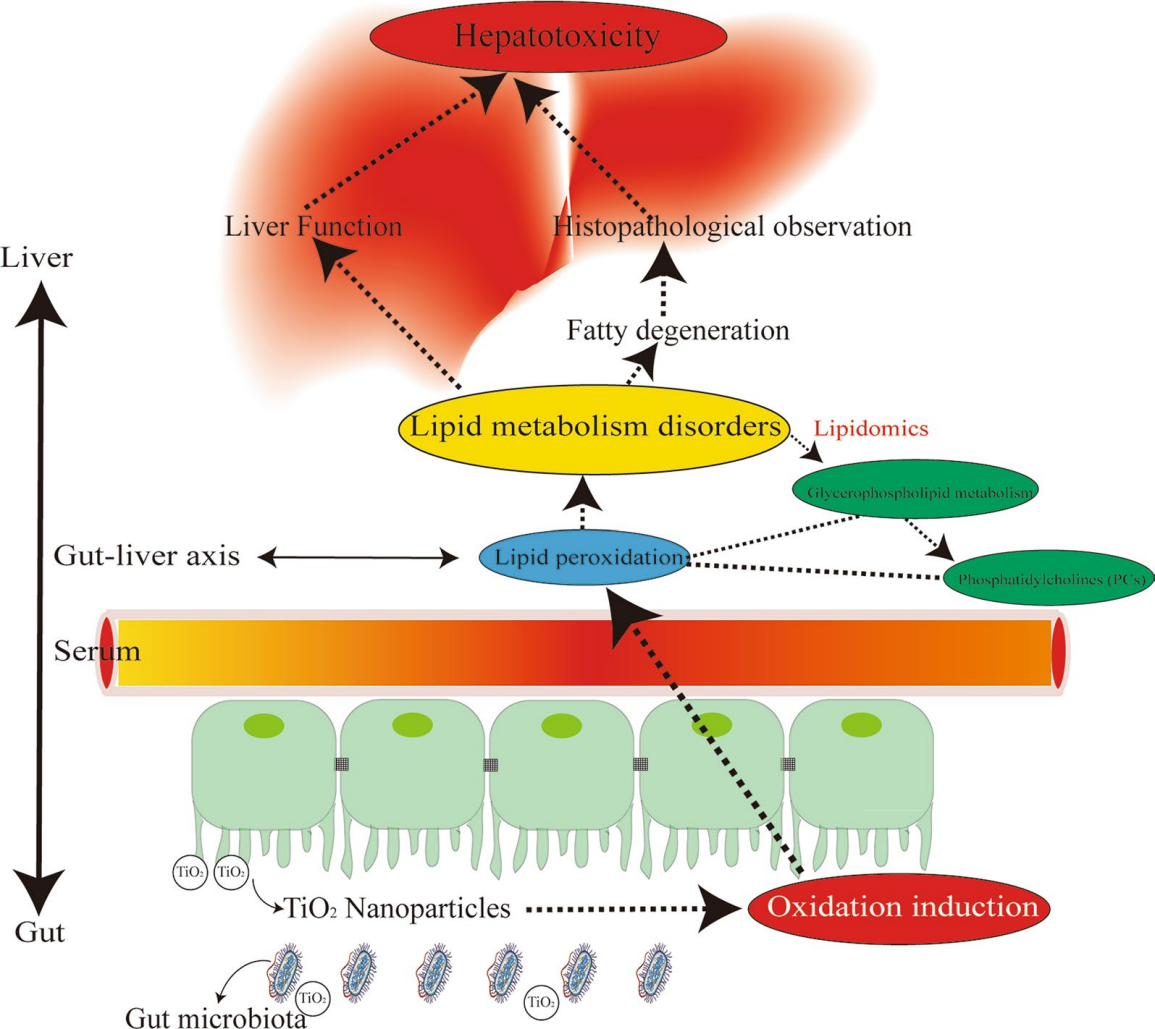
The toxic pathway of hepatotoxicity induced by oral exposure to TiO_2_ NPs through lipid metabolism disorders in gut-liver axis. Due to limited intestinal absorption and strong oxidation induction ability of TiO_2_ NPs, the gut microbiota was presumed to be the original site of oxidative stress and disorders of lipid metabolism. In fact, TiO_2_ NPs changed lipidomic signatures of main organs or systems in the gut-liver axis including liver, serum and gut. The cluster profile from the above biological samples all pointed to the same key metabolic pathway and metabolites, which was glycerophospholipid metabolism and Phosphatidylcholines (PCs), respectively. Lipid peroxidation characterized by the increase of malondialdehyde (MDA) may be the initial step of lipid metabolism disorders, which resulted in hepatic fatty degeneration and liver function changes, leading to hepatotoxicity. Most nanomaterials (NMs) have oxidation induction and antibacterial properties, so this toxic pathway may be primary and universal

However, we found no accumulation of TiO_2_ NPs in the liver through the detection of Ti element (data not shown). Previous study found that the redox balance was destroyed in plasma of rats after oral exposure to 200 mg/kg TiO_2_ NPs for 30 days, even though no obvious accumulation of TiO_2_ NPs was observed [60]. In fact, extremely low absorption of TiO_2_ NPs after long-term and low-dose oral exposure was reported, the proportion of which was estimated to be only approximately 0.02% to 0.1% [24, 26, 27]. Even so, orally ingested TiO_2_ NPs could induce toxicity through indirect ways, such as oxidative stress, inflammation or gut microbiota. Oxidative stress and inflammatory response are often systemic effects that can affect the whole body through the circulatory system. Several publications have demonstrated that the genotoxicity could be induced by nanomaterials through secondary genotoxic mechanism with indirect consequences of inflammation and generation of oxidative species [61–63]. In addition, recent studies found even in the absence of light, TiO_2_ NPs also induced oxidative stress and damaged the intestinal bacteria such as *E. coli *in vitro [33]. And TiO_2_ NPs with smaller particle size produced higher content of intracellular ROS, in line with their greater antibacterial effect [64]. In vivo, studies have demonstrated that gut microbiota disturbance could be caused in rats after oral exposure to TiO_2_ NPs [29–32]. Therefore, it was speculated that orally administered TiO_2_ NPs may interact with gut microbiota through oxidative stress at first, and then affect the gut-liver axis and circulatory system, thereby leading to liver toxicity and other health effects finally (Fig. 8). In these processes, the disorder of lipid metabolism starting from lipid peroxidation should play an important role all the way. Given that most nanomaterials (NMs) have the same characteristics, i.e. oxidation induction and antibacterial [65–68], this toxic pathway may be universal. Attention should be paid to the health hazard of liver injury and gut microbiota disturbance caused by dietary and environmental exposure to NMs.

## Conclusions

In summary, oral exposed TiO_2_ NPs changed lipidomic signatures of main organs or systems in the gut-liver axis including liver, serum and gut. The key metabolic pathways and lipophilic metabolites in the three biological samples were the same, which were glycerophospholipid metabolism and PCs respectively. Lipid peroxidation may be the initial step of lipid metabolism disorder induced by TiO_2_ NPs due to its strong oxidative stress induction ability. The gut microbiota was presumed to be the original site of oxidative stress, which then led to the disorder of lipid metabolism, and resulted in hepatotoxicity through the gut-liver axis. The discovery of universal toxic pathway of oral NMs exposure should be of guiding significance to explore the corresponding measures to reduce toxicity.

## Methods

### Nanoparticle characterization

The titanium dioxide nanoparticles (TiO_2_ NPs) were purchased from Shanghai Macklin Reagent Co. Ltd, China. The size and shape of the particles was characterized by scanning electron microscopy (SEM, Nova, Tecnai F30, FEI Company, Oregon, USA). Energy dispersive X-ray spectroscopy (EDS, Nova_NanoSEM430, FEI Company, Oregon, USA) was used to measure the ratio of Ti to O atoms. The purity of the particles was analyzed by detecting the content of Ti element using inductively coupled plasma mass spectrometry (ICP-MS, IRIS Advantag, TJA, Franklin, MA, USA). The crystal structure of the particles was identified by X-ray powder diffractometry (XRD, PANalytical’s X’Pert PRO, X’Celerator, EA Almelo, Netherlands). The specific surface area (SSA) of the particles was measured according to Brunauer–Emmett–Teller (BET) method (Quantachrome, Autosorb 1, Boynton, FL, USA).

The artificial gastric juice (AGJ, pH 1.2) was prepared using 10 g/L pepsin (3800 units/mg) and 45 mmol/L HCl. The artificial intestinal juice (AIJ; pH 6.8) was made with 10 g/L trypsin (2500 units/mg) and 6.8 g/L KH_2_PO_4_. The pH was adjusted to 6.8 using 0.1 mol/L NaOH. After the TiO_2_ NPs and glucose were dispersed in ultrapure water (H_2_O), AGJ or AIJ to obtain a final concentration of 1 mg/mL TiO_2_ NPs, the suspensions were supersonicated for 15 min to break up aggregates. The particle hydrodynamic diameters and Zeta potentials were tested using the ZetaSizer Nano ZS90 (Malvern Instruments Ltd, Malvern, UK).

### Animal and experimental design

Three-week-old healthy Sprague–Dawley rats were bred and supplied by the Department of Laboratory Animal Science, Peking University Health Science Center. The rats were fed a commercial pellet diet and deionized water ad libitum, and kept in plastic cages at 20 ± 2 ℃ and 50–70% relative humidity with a 12:12 h light–dark cycle. After one week of acclimation, rats were weighed and randomized into experimental and control groups, with 6 male rats in each group.

All experimental rats were provided humane care of the animals. The study was conducted in accordance with the Guiding Principles in the European Union Directive 2010/63/EU for animal experiments, and received approval from the Peking University Institutional Review Board.

Suspensions of TiO_2_ NPs (0, 2, 10, 50 mgkg^−1^ BW) were administered to rats via oral gavage in a volume of 1 mL daily for 90 consecutive days, using new and fresh suspensions of TiO_2_ NPs every day. The animal experiment was carried out according to the guidelines of OECD for the Testing of Chemicals No. 408 Repeated Dose 90-Day Oral Toxicity Study in Rodents. The intragastric doses of TiO_2_ NPs for rats were selected based on the oral intake of TiO_2_ NPs for children using 100 as safety factor, referring to our previous article [39]. Before each treatment of animals, the TiO_2_ NPs were dispersed in ultrapure water and sonicated for 15 min and vortexed for 5 min to maintain uniformity. The symptom and mortality were observed and recorded daily throughout the duration of exposure up to 90 days. The body weight of rats was assessed every 7 days and the food intake of rats was recorded every 3–4 days. After 90 days, animals were weighed and sacrificed. The blood samples were collected from the abdominal aorta. Serum was harvested by centrifuging blood at 3000 rpm (1500 g) for 10 min. The liver tissues and feces were also harvested at the end of the experiment.

### Sample preparation of metabolomic analysis

30.0 mg of liver tissue or vacuum-dried feces was added to 900 μl pre-cooled methanol/water (1:1) solution. Then it was homogenized at 30 000 rpm on ice for 30 s (Homogenized for 10 s, cooled for 30 s, repeated three times) and blended by a vortex for 20 s and stored at – 20 ℃ overnight. And then, the homogenate was centrifuged (16,000 g, 4 C) for 10 min and the supernatant was taken. The supernatant was dried and concentrated in a low temperature vacuum concentrator for 4 h. Re-suspension was carried out by 200 μl methanol/water (1:1) solvent before analysis. Meanwhile, 20 μl of each sample was taken and then divided into three parts after gentle mixing as quality control (Qc) samples, which were prepared for technical repetition to evaluate the stability and repeatability of the experimental instruments and methods.

100 μl serum samples were added to 400 μl pre-cooled chloroform/methanol (2:1) solution. After vortex mixing, use a shaker (Mixmate, Eppendorf, Germany) to further shake for 15 min at 1200 Hz/min. Then it was centrifuged (12,000 rpm, 4 ℃) for 15 min and the lower lipophilic solution was taken. The solution was dried and concentrated in a low temperature vacuum concentrator for 4 h. Re-suspension was carried out by 50 μl acetonitrile/water (1:1) solvent before analysis. Meanwhile, 5 μl of each sample was taken and then divided into three parts after gentle mixing as quality control (Qc) samples.

### Untargeted metabolomics analysis using HPLC–MS

Ultra High Performance Liquid Chromatography-Q-Exactive Orbitrap-High-resolution Mass Spectrometry System (UPLC-QEMS, U3000, Thermo, USA) was used for untargeted metabolomics analysis. The samples were randomly injected after disruption of the order to control the possible impact of instrumental stability fluctuation. Three Qc samples were analyzed before experimental samples, after half of all samples and after all samples, respectively. The QEMS was equipped with an electrospray ionization source (ESI). Fragmentation was achieved by high-energy collision dissociation (HCD). The normalized collision energies were 15, 30 and 45 eV, respectively. The results were measured by positive ion mode and negative ion mode. The mass scanning range was 50–1100 m/z, and the total scanning resolution of parent ions (MS) was 60 K.

### Analysis of metabolomic data

The peaks in the mass spectrum data were collected. The metabolites were identified after relative standard deviation de-noising in the mode of positive and negative ion modes respectively. Then, the missing values were filled up by half of the minimum value. Also, total ion current normalization method was employed in this data analysis. The final dataset containing the information of peak number, sample name and normalized peak area was imported to SIMCA15.0.2 software package (Sartorius Stedim Data Analytics AB, Umea, Sweden) for multivariate analysis. Data was scaled and logarithmic transformed to minimize the impact of both noise and high variance of the variables. After these transformations, PCA (principle component analysis, PCA), an unsupervised analysis that reduces the dimension of the data, was carried out to visualize the distribution and the grouping of the samples. 95% confidence interval in the PCA score plot was used as the threshold to identify potential outliers in the dataset.

In order to visualize group separation and find significantly changed metabolites, supervised orthogonal projections to latent structures- discriminate analysis (OPLS-DA) was applied. Then, a sevenfold cross validation was performed to calculate the value of R2 and Q2. R2 indicates how well the variation of a variable is explained and Q2 means how well a variable could be predicted. To check the robustness and predictive ability of the OPLS-DA model, a 200 times permutations was further conducted. Afterward, the R2 and Q2 intercept values were obtained. Here, the intercept value of Q2 represents the robustness of the model, the risk of overfitting and the reliability of the model, which will be the smaller the better. Furthermore, the value of variable importance in the projection (VIP) of the first principal component in OPLS-DA analysis was obtained. It summarizes the contribution of each variable to the model. The metabolites with VIP > 1 and *p* < 0.05 (student t test) were considered as significantly changed metabolites. In addition, commercial databases including KEGG (Kyoto Encyclopedia of Genes and Genomes) (http://www.genome.jp/kegg/) and MetaboAnalyst (http://www.metaboanalyst.ca/) were used for pathway enrichment analysis.

### Measurement of lipid peroxidation

Malondialdehyde (MDA), a typical lipid peroxidation product, was measured in serum using the Lipid Peroxidation (MDA) Colorimetric Assay Kit (Beijing Leagene Biotechnology Inc, Beijing, China). The MDA in the sample is reacted with Thiobarbituric Acid (TBA) to generate the MDA-TBA adduct. The MDA-TBA adduct can be easily quantified colorimetrically at 532 nm.

### Absolute quantitative lipidomics

With reference to the method of Tu et al. [69], this study applied absolute quantitative lipidomics to verify the key lipid metabolites. 10 μL of the sample was mixed with 190 μL water, and then 480 μL extract solution (Methyl tert butyl ether (MTBE): methanol (MeOH) = 5:1) containing internal standard was added. After vortex, sonication, and centrifugation (3000 rpm for 15 min at 4 ℃), the supernatants were combined and dried in a vacuum concentrator at 4 ℃. Then, the dried samples were reconstituted in 100 μL of resuspension buffer (Dichloromethane (DCM): MeOH = 1:1) by sonication on ice for 10 min. The constitution was then centrifuged at 12,000 rpm for 15 min at 4 ℃, and 30 μL of supernatant was transferred to a fresh glass vial for LC/MS analysis. The UHPLC separation was carried out using a SCIEX ExionLC series UHPLC system equipped with a ACQUITY UPLC HSS T3 1.8 μm 2.1 * 100 mm column. The mobile phase A consisted of 40% water, 60% acetonitrile, and 10 mmol/L ammonium formate. The mobile phase B consisted of 10% acetonitrile and 90% isopropanol, and 10 mmol/L ammonium formate. The column temperature was 40 ℃. The auto-sampler temperature was 6 ℃, and the injection volume was 2 μL. AB Sciex QTrap 6500 + mass spectrometer was applied for assay development. Typical ion source parameters were: IonSpray Voltage: + 5500/− 4500 V, Curtain Gas: 40 psi, Temperature: 350℃, Ion Source Gas 1:50 psi, Ion Source Gas 2: 50 psi. Skyline 20.1 Software was employed for the quantification of the target compounds. The absolute content of individual lipids corresponding to the internal standard (IS) was calculated on the basis of peaks area and actual concentration of the identical lipid class IS.

### Statistical analysis

Methods of statistical analysis for data of metabolomics were described above. Other data were expressed as means ± SD and analyzed with SPSS 20.0. When the homogeneity of variance is satisfied, One-way variance (ANOVA) with LSD test was applied to evaluate the statistical significance of differences between the experimental groups and the control. When not satisfied, the differences among groups were compared by nonparametric Kruskal Wallis method with Steel Dwass test. A *p* value of less than 0.05 was considered to be statistically significant.

## Supplementary Information

**Additional file 1: Table S1**. Hepatic differential lipophilic metabolites with good matches between the treated group (50 mg/kg) and the control group. **Table S2**. Serum differential lipophilic metabolites with good matches between the treated group (50 mg/kg) and the control group. **Table S3**. Feces differential lipophilic metabolites with good matches between the treated group (50 mg/kg) and the control group. **Table S4**. Differential phosphatidylcholines (PCs) in liver, serum and feces between the treated group (50 mg/kg) and the control group. **Figure S1**. KEGG pathway map of glycerophospholipid metabolism. **Figure S2**. Score scatter plot for principal component analysis (PCA) model with total lipophilic metabolites and quality control (QC) samples. **Figure S3**. Permutation test of orthogonal projections to latent structures-discriminant analysis (OPLS-DA) model.

## Data Availability

The datasets used and/or analyzed during the current study are available from the corresponding author on reasonable request.

## Abbreviations

TiO_2_: Titanium dioxide
NPs: Nanoparticles
SEM: Scanning electron microscopy
DLS: Dynamic light scattering
EDS: X-ray spectroscopy
ICP-MS: Inductively coupled plasma mass spectrometry
XRD: X-ray powder diffractometry
BET: Brunauer–Emmett–Teller
AGJ: Artificial gastric juice
AIJ: Artificial intestinal juice
TBIL: Total bilirubin
Qc: Quality control
UPLC-QEMS: Ultra high performance liquid chromatography-Q-exactive orbitrap-high-resolution mass spectrometry system
PCA: Principal component analysis
OPLS-DA: Orthogonal projection to latent structure discriminant analysis
VIP: Variable importance in the projection
KEGG: Kyoto encyclopedia of genes and genomes
MDA: Malondialdehyde
