# Supplementary material for: Landscape of lipidomic metabolites in gut-liver axis of Sprague–Dawley rats after oral exposure to titanium dioxide nanoparticles
Source: Part Fibre Toxicol. 2022 Aug 3;19:53. doi: 10.1186/s12989-022-00484-9 (PMC9351087; doi:10.1186/s12989-022-00484-9)
Supplement: Supplementary file 1 — Additional file 1: Table S1. Hepatic differential lipophilic metabolites with good matches between the treated group (50 mg/kg) and the control group. Table S2. Serum differential lipophilic metabolites with good matches between the treated group (50 mg/kg) and the control group. Table S3. Feces differential lipophilic metabolites with good matches between the treated group (50 mg/kg) and the control group. Table S4. Differential phosphatidylcholines (PCs) in liver, serum and feces between the treated group (50 mg/kg) and the control group. Figure S1. KEGG pathway map of glycerophospholipid metabolism. Figure S2. Score scatter plot for principal component analysis (PCA) model with total lipophilic metabolites and quality control (QC) samples. Figure S3. Permutation test of orthogonal projections to latent structures-discriminant analysis (OPLS-DA) model. [file 12989_2022_484_MOESM1_ESM.pdf]

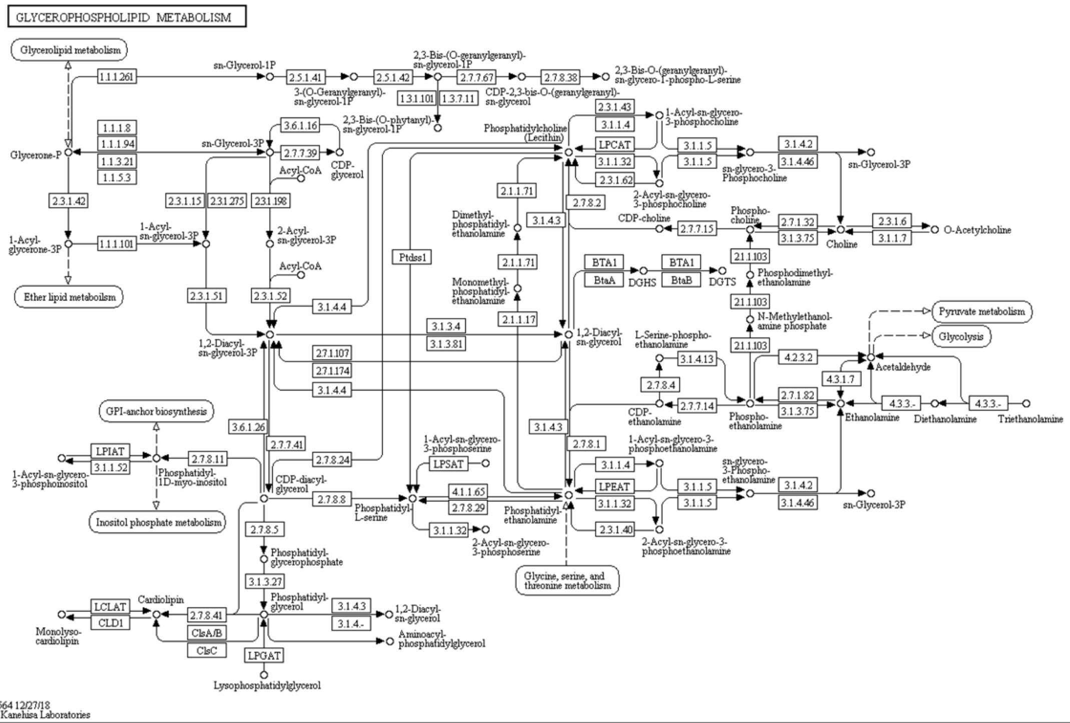

Figure S1. KEGG pathway map of glycerophospholipid metabolism.

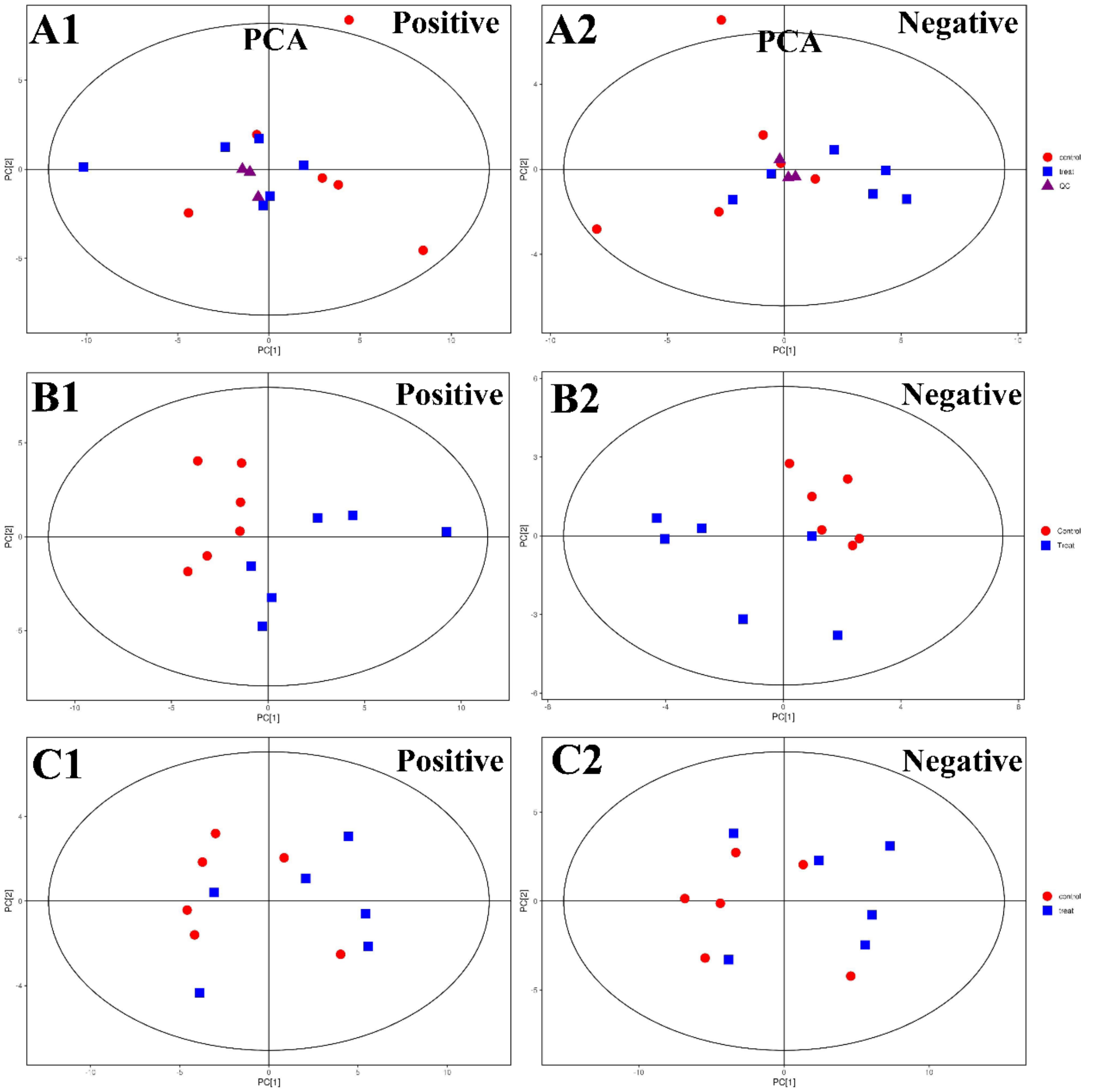

Figure S2. Score scatter plot for principal component analysis (PCA) model with total lipophilic metabolites and quality control (QC) samples. Samples included liver tissue (A1 and A2), serum (B1 and B2) and feces (C1 and C2). The X-axis PC[1] and Y-axis PC[2] represent the scores of the first and second principal components respectively. Most samples are in 95% confidence interval (Hotelling's t-squared ellipse). PCA plots showed the overall difference in metabolites between the treated group and the control group.

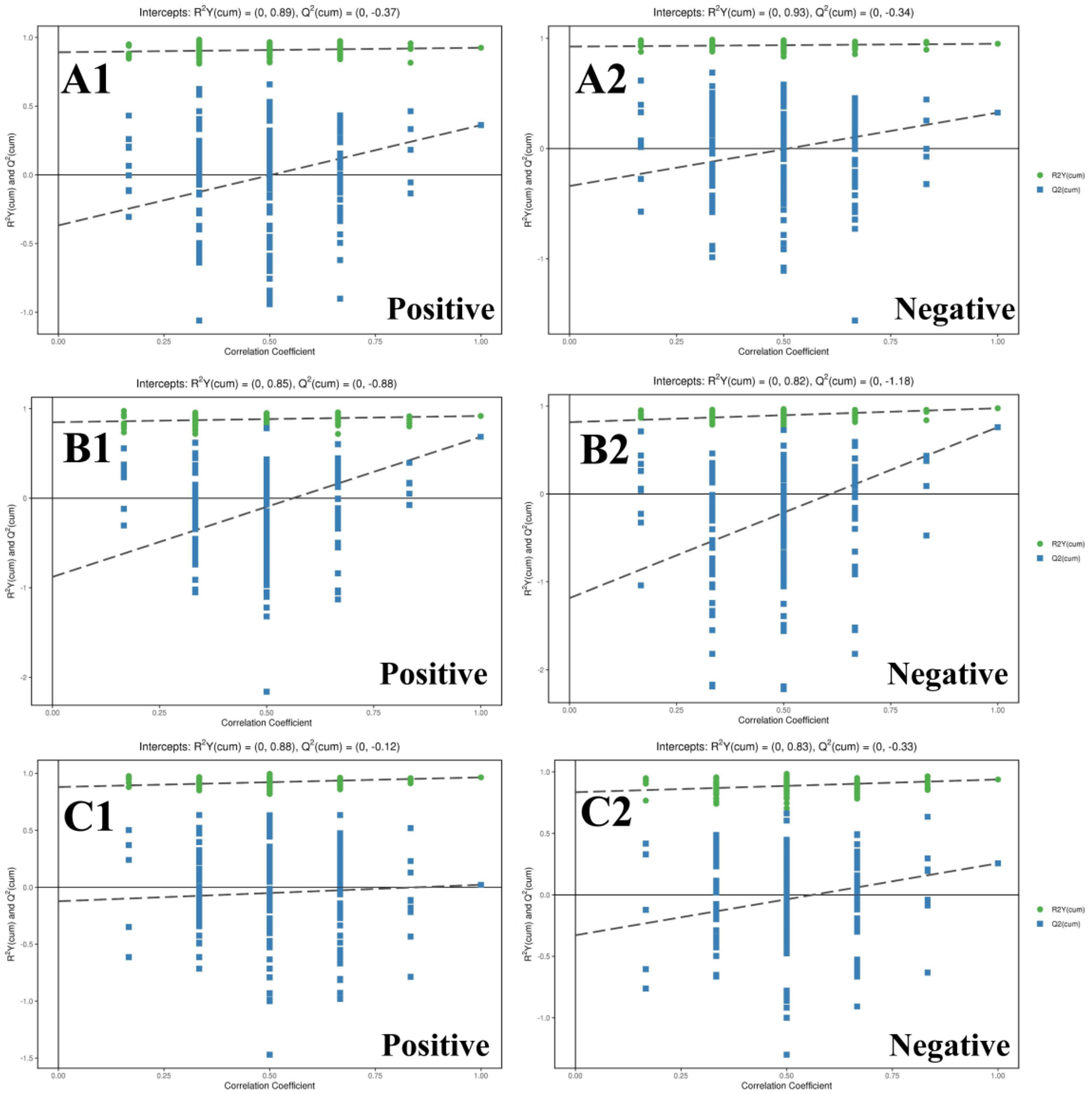

Figure S3. Permutation test of orthogonal projections to latent structures-discriminant analysis (OPLS-DA) model. Samples included liver tissue (A1 and A2), serum (B1 and B2) and feces (C1 and C2). The model parameter of R^2Y was very close to 1, indicating that the established model conforms to the real situation of sample data. The model parameter of Q^2 is relatively close to 1, indicating that the model can better explain the differences between the two groups of samples.

Table S1. Hepatic differential lipophilic metabolites with good matches between the treated group (50 mg/kg) and the control group.

No.	Mode	MS2 name	MS2 score	Retention time, RT (min)	molecular mass	VIP	P value	FDR	Fold Change	log_2 Fold Change
1	positive	DG(18:2(9Z,12Z)/18:3(9Z,12Z,15Z)/0:0)[iso2]	1.000	688.556	614.561	1.785	0.037	0.553	1.374	0.458
2	positive	PS(13:0/12:0)	0.997	154.508	637.416	1.877	0.039	0.553	0.737	-0.441
3	positive	Pregnanetriol	0.996	668.717	337.272	1.822	0.041	0.553	1.444	0.530
4	positive	TG(18:3(9Z,12Z,15Z)/20:3(8Z,11Z,14Z)/22:5(7Z,10Z,13Z,16Z,19Z))[iso6]	0.995	824.285	952.827	2.114	0.018	0.553	1.705	0.770
5	positive	Nervonic Ceramide	0.995	716.279	649.564	1.941	0.019	0.553	0.804	-0.315
6	positive	Ubiquinone 8	0.995	698.598	727.565	1.833	0.006	0.553	1.418	0.504
7	positive	Lyso-	0.994	135.170	482.358	1.681	0.026	0.553	2.308	1.206
8	positive	TG(17:0/17:2(9Z,12Z)/17:2(9Z,12Z))[iso3]	0.994	719.883	841.712	1.888	0.040	0.553	0.812	-0.301
9	positive	1-(14-methyl-pentadecanoyl)-2-(8-[3]-ladderane-octanyl)-sn-glycerol	0.991	691.708	603.538	1.665	0.044	0.553	1.355	0.438
10	positive	PC(22:6(4Z,7Z,10Z,13Z,16Z,19Z)/20:3(5Z,8Z,11Z))	0.989	463.057	856.571	2.125	0.017	0.553	1.418	0.503
11	positive	Met Asn Gly	0.986	41.842	321.130	1.832	0.029	0.553	0.642	-0.639
12	positive	PC(22:6(4Z,7Z,10Z,13Z,16Z,19Z)/20:4(5Z,8Z,11Z,14Z))	0.984	464.101	854.565	2.078	0.019	0.553	1.398	0.483
13	positive	Lys Lys Lys Phe	0.981	165.453	550.385	1.546	0.043	0.553	0.754	-0.407
14	positive	N-(5-hydroxy-pentyl) arachidonoyl amine	0.979	74.041	390.336	2.248	0.005	0.553	1.884	0.914
15	positive	PC(O-16:1(11Z)/2:0)	0.979	113.157	522.352	2.106	0.012	0.553	0.696	-0.524
16	positive	Cer(d18:0/24:0(2OH))	0.968	733.905	668.651	1.743	0.010	0.553	0.818	-0.290
17	positive	2-[2-[(3E,5E)-7-hydroxy-3,5-dimethyl-2-oxoocta-3,5-dienyl]-6-oxooxan-4-yl]acetamide	0.966	50.529	323.198	1.740	0.049	0.553	0.660	-0.600
18	positive	2-[[(2R)-2,3-dihydroxypropoxy]-hydroxyphosphoryl]oxyethyl-trimethylazanium	0.942	41.408	257.146	2.383	0.007	0.553	0.236	-2.081
19	positive	PC(20:3(8Z,11Z,14Z)/0:0)	0.931	85.344	546.345	1.857	0.031	0.553	0.710	-0.494
20	positive	PI(17:0/20:4(5Z,8Z,11Z,14Z))	0.864	592.649	891.572	1.625	0.048	0.553	0.649	-0.623
21	positive	2,3-dihydroxypropyl octadecanoate	0.842	258.192	359.314	1.631	0.030	0.553	1.224	0.291
22	positive	DG(12:0/17:2(9Z,12Z)/0:0)[iso2]	0.823	659.443	523.571	2.315	0.004	0.553	0.700	-0.514
23	positive	PC(O-18:2(9Z,12Z)/2:0)	0.806	120.696	548.370	1.974	0.014	0.553	0.662	-0.595
24	positive	ML-9	0.729	58.400	325.180	2.367	0.021	0.553	0.452	-1.144
25	positive	[2,5,7,8-tetramethyl-2-(4,8,12-trimethyltridecyl)-3,4-dihydrochromen-6-yl] acetate	0.700	252.291	490.425	2.261	0.003	0.553	2.126	1.088
26	positive	Spisulosine	0.661	40.679	287.084	1.839	0.040	0.553	0.386	-1.374
27	positive	TG(14:1(9Z)/15:1(9Z)/18:4(6Z,9Z,12Z,15Z))[iso6]	0.562	655.219	781.083	1.761	0.042	0.553	1.437	0.523
28	positive	PS(18:1(9Z)/0:0)	0.519	113.157	523.356	2.098	0.012	0.553	0.699	-0.517
29	positive	Nandrolone phenpropionate	0.413	109.329	407.254	1.647	0.041	0.553	0.612	-0.709
30	positive	1alpha,25-dihydroxy-3-deoxy-3-thiavitamin D3 3-oxide / 1alpha,25-dihydroxy-3-deoxy-3-thiacholecalciferol 3-oxide	0.362	161.668	435.285	1.847	0.006	0.553	0.587	-0.767

Table S1. Hepatic differential lipophilic metabolites with good matches between the treated group (50 mg/kg) and the control group. (**Continued**)

No.	Mode	MS2 name	MS2 score	Retention time, RT (min)	molecular mass	VIP	P value	FDR	Fold Change	log_2 Fold Change
31	negative	20-Hydroxy-PGF2a	0.981	49.662	369.230	1.751	0.040	0.549	0.630	-0.667
32	negative	[3-hexadecanoyloxy-2-[(4Z,7Z)-octadeca-4,7-dienoyl]oxypropyl] 2-(trimethylazaniumyl)ethyl phosphate	0.865	641.220	817.581	1.681	0.045	0.549	0.820	-0.287
33	negative	[(2R)-1-[2-aminoethoxy(hydroxy)phosphoryl]oxy-3-hexadecanoyloxypropan-2-yl] (9Z,12Z)-octadeca-9,12-dienoate	0.757	633.782	714.512	1.930	0.019	0.549	1.176	0.234
34	negative	[3-hexadecanoyloxy-2-[(4Z,7Z,10Z,13Z)-icosa-4,7,10,13-tetraenoyl]oxypropyl] 2-(trimethylazaniumyl)ethyl phosphate	0.732	643.242	839.586	1.826	0.029	0.549	1.283	0.360

DG: Diacylglycerol; PS: Phosphatidylserine; TG: Triacylglycerol; PC: Phosphatidylcholine; PI: phosphatidylinositol. Red represents the concentration of metabolite in the treat group is higher than the control group, and blue represents the concentration of metabolite in the treat group is lower than the control group.

Table S2. Serum differential lipophilic metabolites with good matches between the treated group (50 mg/kg) and the control group.

No.	Mode	MS2 name	MS2 score	Retention time, RT (min)	molecular mass	VIP	P value	FDR	Fold Change	log_2 Fold Change
1	positive	Val Arg Ser	1.000	45.005	361.218	1.686	0.032	0.170	1.865	0.899
2	positive	12-HETE	0.999	80.200	303.230	1.584	0.003	0.081	0.449	-1.154
3	positive	DG(16:1(9Z)/22:5(7Z,10Z,13Z,16Z,19Z)/0:0)[iso2]	0.997	670.377	641.509	1.587	0.046	0.180	0.564	-0.825
4	positive	PE(22:4(7Z,10Z,13Z,16Z)/21:0)	0.996	667.409	838.628	1.082	0.027	0.166	1.292	0.369
5	positive	PC(22:4(7Z,10Z,13Z,16Z)/14:0)	0.995	609.008	782.567	1.794	0.001	0.055	1.550	0.632
6	positive	PC(22:4(7Z,10Z,13Z,16Z)/16:0)	0.993	650.851	810.599	1.862	0.000	0.007	1.347	0.430
7	positive	PE-NMe2(16:0/20:4(5Z,8Z,11Z,14Z))[S]	0.992	557.842	768.549	1.566	0.009	0.128	1.853	0.890
8	positive	PC(O-18:2(9Z,12Z)/0:0)[U]	0.991	169.602	506.358	1.352	0.032	0.170	0.836	-0.258
9	positive	PC(19:1(9Z)/17:2(9Z,12Z))	0.991	618.823	784.576	1.328	0.023	0.162	1.178	0.236
10	positive	1-heptadecanoyl-sn-glycero-3-phosphocholine	0.989	135.903	510.354	1.503	0.007	0.117	0.738	-0.439
11	positive	PC(22:1(11Z)/12:0)	0.989	648.742	760.577	1.308	0.042	0.176	1.153	0.206
12	positive	TG(18:0/18:4(6Z,9Z,12Z,15Z)/18:4(6Z,9Z,12Z,15Z))[iso3]	0.988	786.576	875.706	1.721	0.021	0.159	0.575	-0.797
13	positive	PC(20:1(11Z)/12:0)	0.988	610.953	732.550	1.077	0.028	0.167	1.383	0.468
14	positive	PC(24:1(15Z)/18:4(6Z,9Z,12Z,15Z))	0.988	670.534	864.644	1.892	0.000	0.012	2.020	1.015
15	positive	PI(22:0/21:0)	0.987	767.352	965.695	1.446	0.032	0.170	1.209	0.274
16	positive	PC(O-16:0/2:0)	0.987	169.244	524.368	1.746	0.003	0.087	0.829	-0.270
17	positive	PC(18:0/14:0)	0.985	652.853	734.566	1.709	0.009	0.132	1.360	0.443
18	positive	PC(18:2(9Z,12Z)/22:1(11Z))	0.984	672.789	840.639	1.276	0.007	0.119	1.401	0.486
19	positive	Ala Arg Pro	0.984	47.489	343.210	1.176	0.028	0.167	0.601	-0.734
20	positive	TG(12:0/20:4(5Z,8Z,11Z,14Z)/22:3(10Z,13Z,16Z))[iso6]	0.984	802.383	877.723	1.561	0.022	0.161	0.718	-0.477
21	positive	TG(12:0/20:3(8Z,11Z,14Z)/22:3(10Z,13Z,16Z))[iso6]	0.983	818.654	879.738	1.653	0.015	0.148	0.569	-0.814
22	positive	PC(24:1(15Z)/18:3(6Z,9Z,12Z))	0.981	690.242	866.660	1.605	0.002	0.074	1.551	0.633
23	positive	3alpha-Acetomethoxy-11alpha-oxo-12-ursen-24-oic acid	0.981	169.399	527.377	1.735	0.005	0.101	0.802	-0.318
24	positive	TG(18:2(9Z,12Z)/18:0/18:3(9Z,12Z,15Z))[iso6]	0.981	837.016	881.751	1.559	0.027	0.166	0.619	-0.692
25	positive	PC(22:1(11Z)/20:5(5Z,8Z,11Z,14Z,17Z))	0.980	660.084	862.626	1.321	0.031	0.169	1.219	0.285
26	positive	N-stearoyl tyrosine	0.979	64.735	448.336	1.856	0.004	0.094	1.541	0.624
27	positive	PC(22:2(13Z,16Z)/17:2(9Z,12Z))	0.977	662.109	824.613	1.664	0.001	0.068	1.562	0.643
28	positive	PC(20:3(8Z,11Z,14Z)/17:1(9Z))	0.974	636.918	796.584	1.302	0.048	0.181	1.349	0.432
29	positive	Oleamide	0.972	163.431	282.278	1.157	0.035	0.172	0.668	-0.582
30	positive	PE(22:1(11Z)/20:2(11Z,14Z))	0.972	665.540	826.624	1.279	0.006	0.115	1.347	0.430
31	positive	11-cis retro-gamma-retinal	0.970	79.854	285.220	1.578	0.003	0.084	0.453	-1.142
32	positive	PC(O-16:0/3:1(2E))	0.967	139.567	536.369	1.846	0.000	0.038	0.644	-0.634
33	positive	Estradiol-17-phenylpropionate	0.960	50.435	405.237	1.899	0.012	0.142	1.682	0.750
34	positive	LysoPE(20:1(11Z)/0:0)	0.959	97.558	508.339	1.649	0.002	0.080	0.688	-0.540
35	positive	C22 Sulfatide	0.956	59.585	864.623	1.784	0.029	0.168	2.097	1.068

Table S2. Serum differential lipophilic metabolites with good matches between the treated group (50 mg/kg) and the control group. (**Continued**)

No.	Mode	MS2 name	MS2 score	Retention time, RT (min)	molecular mass	VIP	P value	FDR	Fold Change	log_2 Fold Change
36	positive	PE-NMe(16:0/16:0)	0.954	605.062	706.525	1.817	0.000	0.036	1.366	0.450
37	positive	2-amino-octadecanoic acid	0.954	148.657	300.289	1.709	0.006	0.112	0.708	-0.499
38	positive	PC(20:3(8Z,11Z,14Z)/0:0)	0.940	91.676	546.348	1.305	0.009	0.132	0.625	-0.679
39	positive	LysoPC(P-16:0)	0.931	128.628	480.341	1.399	0.008	0.125	0.775	-0.368
40	positive	lysoPC(26:1(5Z))	0.923	455.455	634.477	1.612	0.032	0.170	1.336	0.418
41	positive	Tricosanamide	0.905	738.567	636.626	1.042	0.037	0.173	1.484	0.569
42	positive	Lauryl diethanolamide	0.892	66.820	288.249	1.636	0.001	0.066	0.567	-0.818
43	positive	PE(22:0/0:0)	0.889	199.073	538.386	1.345	0.014	0.147	0.753	-0.410
44	positive	PC(22:6(4E,7E,10E,13E,16E,19E)/0:0)[U]	0.888	78.873	568.337	1.400	0.013	0.145	0.730	-0.454
45	positive	Lyso-PAF C-18	0.860	205.212	510.391	1.813	0.000	0.036	0.610	-0.714
46	positive	Oleoyl Ethyl Amide	0.858	240.921	310.309	1.375	0.039	0.174	0.460	-1.120
47	positive	Asn Arg Arg Val	0.855	84.199	544.336	1.388	0.008	0.126	0.897	-0.156
48	positive	PC(22:5(4Z,7Z,10Z,13Z,16Z)/20:2(11Z,14Z))	0.855	640.460	860.611	1.406	0.020	0.158	1.352	0.435
49	positive	Enigmol	0.855	99.634	302.304	1.245	0.048	0.181	0.705	-0.504
50	positive	PC(22:6(4Z,7Z,10Z,13Z,16Z,19Z)/19:1(9Z))	0.853	665.925	846.605	1.561	0.009	0.130	1.398	0.483
51	positive	PC(22:6(4Z,7Z,10Z,13Z,16Z,19Z)/19:0)	0.836	656.395	848.622	1.266	0.039	0.174	1.244	0.315
52	positive	C22-OH Sulfatide	0.823	53.259	880.618	1.915	0.031	0.169	2.113	1.079
53	positive	PC(24:1(15Z)/22:4(7Z,10Z,13Z,16Z))	0.820	717.793	920.699	2.024	0.000	0.030	2.024	1.017
54	positive	sn-Glycero-3-phosphocholine	0.811	42.073	258.110	1.290	0.045	0.179	0.577	-0.793
55	positive	Lys Thr Arg Arg	0.792	196.008	560.367	1.529	0.015	0.149	0.622	-0.686
56	positive	PC(24:0/20:5(5Z,8Z,11Z,14Z,17Z))	0.743	693.656	892.673	1.962	0.000	0.007	2.779	1.475
57	positive	PC(P-15:0/0:0)	0.720	209.325	466.328	1.811	0.001	0.045	0.632	-0.661
58	positive	PC(22:6(4Z,7Z,10Z,13Z,16Z,19Z)/15:1(9Z))	0.712	557.842	790.532	1.592	0.004	0.089	2.013	1.009
59	positive	Oleoyl Ethanolamide-d2	0.690	224.540	328.319	1.522	0.042	0.177	0.760	-0.396
60	positive	Scytophycin D	0.690	577.599	844.524	1.519	0.007	0.118	1.235	0.304
61	positive	PC(O-16:0/15:0)	0.670	609.938	706.571	1.349	0.011	0.138	1.203	0.267
62	positive	CerP(d18:1/12:0)	0.619	216.353	562.416	1.180	0.037	0.173	0.821	-0.285
63	positive	PC(18:2(9Z,12Z)/0:0)	0.600	90.319	520.337	1.294	0.015	0.150	0.776	-0.366
64	positive	Gentamicin C2b	0.572	142.638	464.313	1.373	0.013	0.144	0.746	-0.423
65	positive	PC(19:3(10Z,13Z,16Z)/0:0)	0.569	139.919	532.334	1.505	0.012	0.141	0.645	-0.633
66	positive	1-Oleoylglycerophosphocholine	0.547	117.136	522.352	1.646	0.001	0.061	0.762	-0.392
67	positive	Leu Leu Lys Glu	0.534	132.836	502.328	1.474	0.017	0.154	0.624	-0.680
68	positive	4S,5S-antillatoxin A	0.512	136.621	504.341	1.690	0.013	0.145	0.588	-0.766
69	positive	Met Arg Arg Gln	0.464	80.042	590.318	1.330	0.018	0.154	0.758	-0.399
70	negative	15-deoxy-delta-12,14-PGJ2-d4	0.882	77.904	319.222	1.644	0.001	0.007	0.402	-1.314

PC: Phosphatidylcholine; PE: Phosphatidylethanolamine. Red represents the concentration of metabolite in the treat group is higher than the control group, and blue represents the concentration of metabolite in the treat group is lower than the control group.

Table S3. Feces differential lipophilic metabolites with good matches between the treated group (50 mg/kg) and the control group.

No.	Mode	MS2 name	MS2 score	Retention time, RT (min)	molecular mass	VIP	P value	FDR	Fold Change	log_2 Fold Change
1	positive	PC(18:3(6Z,9Z,12Z)/16:0)	0.924	567.809	756.557	1.744	0.014	0.500	1.724	0.786
2	positive	PS(16:0/16:0)	0.979	568.440	735.580	1.944	0.011	0.473	1.435	0.521
3	positive	TG(18:2(9Z,12Z)/16:0/20:5(5Z,8Z,11Z,14Z,17Z))	0.482	736.419	876.732	2.338	0.002	0.236	2.557	1.355
4	negative	LysoPA(18:1(9Z)/0:0)	0.925	135.437	434.268	1.911	0.015	0.211	0.657	-0.606
5	negative	PE(16:0/18:2(9Z,12Z))	0.707	537.955	715.481	1.703	0.006	0.198	0.270	-1.887
6	negative	JWH 007-d9	0.686	41.976	362.935	1.270	0.024	0.215	3.232	1.692
7	negative	1-heptadecanoyl-sn-glycero-3-phosphocholine	0.524	289.999	508.348	1.592	0.042	0.217	1.251	0.323

PC: Phosphatidylcholine; PS: Phosphatidylserine; TG: Triacylglycerol; PE: Phosphatidylethanolamine. Red represents the concentration of metabolite in the treat group is higher than the control group, and blue represents the concentration of metabolite in the treat group is lower than the control group.

Table S4. Differential phosphatidylcholines (PCs) in liver, serum and feces between the treated group (50 mg/kg) and the control group.

Sample	MS2 name	MS2 score	Retention time, RT (min)	molecular mass, mz	Mean control	Mean treat	Fold Change
liver	PC(22:6(4Z,7Z,10Z,13Z,16Z,19Z)/20:3(5Z,8Z,11Z))	0.989	463.1	856.6	0.00009	0.00013	1.418
liver	PC(22:6(4Z,7Z,10Z,13Z,16Z,19Z)/20:4(5Z,8Z,11Z,14Z))	0.984	464.1	854.6	0.00053	0.00073	1.398
liver	PC(O-16:1(11Z)/2:0)	0.979	113.2	522.4	0.00707	0.00492	0.696
liver	PC(20:3(8Z,11Z,14Z)/0:0)	0.931	85.3	546.3	0.00261	0.00185	0.710
liver	PC(O-18:2(9Z,12Z)/2:0)	0.806	120.7	548.4	0.00065	0.00043	0.662
serum	PC(22:4(7Z,10Z,13Z,16Z)/14:0)	0.995	609.0	782.6	0.01964	0.03044	1.550
serum	PC(22:4(7Z,10Z,13Z,16Z)/16:0)	0.993	650.9	810.6	0.01619	0.02182	1.347
serum	PC(O-18:2(9Z,12Z)/0:0)[U]	0.991	169.6	506.4	0.00009	0.00008	0.836
serum	PC(19:1(9Z)/17:2(9Z,12Z))	0.991	618.8	784.6	0.00747	0.00879	1.178
serum	PC(22:1(11Z)/12:0)	0.989	648.7	760.6	0.00555	0.00640	1.153
serum	PC(20:1(11Z)/12:0)	0.988	611.0	732.6	0.00027	0.00037	1.383
serum	PC(24:1(15Z)/18:4(6Z,9Z,12Z,15Z))	0.988	670.5	864.6	0.00009	0.00018	2.020
serum	PC(O-16:0/2:0)	0.987	169.2	524.4	0.05050	0.04189	0.829
serum	PC(18:0/14:0)	0.985	652.9	734.6	0.00098	0.00134	1.360
serum	PC(18:2(9Z,12Z)/22:1(11Z))	0.984	672.8	840.6	0.00021	0.00029	1.401
serum	PC(24:1(15Z)/18:3(6Z,9Z,12Z))	0.981	690.2	866.7	0.00009	0.00013	1.551
serum	PC(22:1(11Z)/20:5(5Z,8Z,11Z,14Z,17Z))	0.980	660.1	862.6	0.00015	0.00018	1.219
serum	PC(22:2(13Z,16Z)/17:2(9Z,12Z))	0.977	662.1	824.6	0.00066	0.00102	1.562
serum	PC(20:3(8Z,11Z,14Z)/17:1(9Z))	0.974	636.9	796.6	0.00176	0.00238	1.349
serum	PC(O-16:0/3:1(2E))	0.967	139.6	536.4	0.00024	0.00015	0.644
serum	PC(20:3(8Z,11Z,14Z)/0:0)	0.940	91.7	546.3	0.00170	0.00106	0.625
serum	PC(22:6(4E,7E,10E,13E,16E,19E)/0:0)[U]	0.888	78.9	568.3	0.00228	0.00166	0.730
serum	PC(22:5(4Z,7Z,10Z,13Z,16Z)/20:2(11Z,14Z))	0.855	640.5	860.6	0.00028	0.00038	1.352
serum	PC(22:6(4Z,7Z,10Z,13Z,16Z,19Z)/19:1(9Z))	0.853	665.9	846.6	0.00010	0.00014	1.398
serum	PC(22:6(4Z,7Z,10Z,13Z,16Z,19Z)/19:0)	0.836	656.4	848.6	0.00017	0.00021	1.244
serum	PC(24:1(15Z)/22:4(7Z,10Z,13Z,16Z))	0.820	717.8	920.7	0.00003	0.00006	2.024
serum	PC(24:0/20:5(5Z,8Z,11Z,14Z,17Z))	0.743	693.7	892.7	0.00005	0.00014	2.779
serum	PC(P-15:0/0:0)	0.720	209.3	466.3	0.00006	0.00004	0.632
serum	PC(22:6(4Z,7Z,10Z,13Z,16Z,19Z)/15:1(9Z))	0.712	557.8	790.5	0.00002	0.00004	2.013
serum	PC(O-16:0/15:0)	0.670	609.9	706.6	0.00034	0.00040	1.203
serum	PC(18:2(9Z,12Z)/0:0)	0.600	90.3	520.3	0.01228	0.00953	0.776
serum	PC(19:3(10Z,13Z,16Z)/0:0)	0.569	139.9	532.3	0.00048	0.00031	0.645
feces	PC(18:3(6Z,9Z,12Z)/16:0)	0.924	567.8	756.6	0.00062	0.00107	1.724
